# Single-cell transcriptome profiling of the human endometrium of patients with recurrent implantation failure

**DOI:** 10.7150/thno.74053

**Published:** 2022-09-06

**Authors:** Zhen-Zhen Lai, Yun Wang, Wen-Jie Zhou, Zhou Liang, Jia-Wei Shi, Hui-Li Yang, Feng Xie, Wei-Dong Chen, Rui Zhu, Ce Zhang, Jie Mei, Jian-Yuan Zhao, Jiang-Feng Ye, Tao Zhang, Ming-Qing Li

**Affiliations:** 1 Laboratory for Reproductive Immunology, Hospital of Obstetrics and Gynecology, Fudan University, Shanghai 200080, People's Republic of China.; 2 NHC Key Lab of Reproduction Regulation, Shanghai Institute for Biomedical and Pharmaceutical Technologies, Fudan University, Shanghai 201203, People's Republic of China.; 3 Department of Assisted Reproduction, Shanghai Ninth People's Hospital Affiliated Shanghai JiaoTong University School of Medicine, Shanghai 200011, People's Republic of China.; 4 Center of Reproductive Medicine of Ruijin Hospital, Shanghai Jiao Tong University School of Medicine, Shanghai 200025, People's Republic of China; 5 Shanghai Key Laboratory of Female Reproductive Endocrine Related Diseases, Hospital of Obstetrics and Gynecology, Fudan University, Shanghai 200080, People's Republic of China.; 6 Center for Diagnosis and Treatment of Cervical and Uterine Diseases, Hospital of Obstetrics and Gynecology, Fudan University, Shanghai 200011, People's Republic of China.; 7 NovelBio Bio-Pharm Technology Co., Ltd, Shanghai 201112, People's Republic of China.; 8 Center for Human Reproduction and Genetics, Affiliated Suzhou Hospital of Nanjing Medical University, Suzhou Municipal Hospital, Gusu School, Nanjing Medical University, Suzhou 215002, People's Republic of China.; 9 State Key Laboratory of Reproductive Medicine, Nanjing Medical University, Nanjing 210029, People's Republic of China.; 10 Reproductive Medicine Center, Department of Obstetrics and Gynecology, Nanjing Drum Tower Hospital, The Affiliated Hospital of Nanjing University Medicine School, Nanjing, 210000, People's Republic of China.; 11 State Key Laboratory of Genetic Engineering, Collaborative Innovation Center for Genetics and Development, School of Life Sciences, Fudan University, Shanghai 200433, People's Republic of China.; 12 Institute of Metabolism and Integrative Biology (IMIB), School of Life Sciences, Fudan University, Shanghai, 200433, People's Republic of China.; 13 Division of Obstetrics and Gynecology, KK Women's and Children's Hospital, 229899, Singapore.; 14 Assisted Reproductive Technology Unit, Department of Obstetrics and Gynecology, Faculty of Medicine, Chinese University of Hong Kong, Hong Kong, People's Republic of China.

**Keywords:** recurrent implantation failure, endometrial receptivity, endometrial fibroblast-like cell, epithelial cell, NK cell

## Abstract

**Introduction:** Despite great advances in assisted reproductive technology (ART), recurrent implantation failure (RIF) cannot be effectively avoided. Notably, cellular characteristics and communication that regulate endometrial receptivity and differentiation, and its disorders in RIF at window of implantation (WOI) remain rudimentary.

**Objectives:** In this study, we profiled the endometrial cells present at the WOI timing in RIF patients and healthy controls using single-cell RNA sequencing (scRNA-seq) and provided a detailed molecular and cellular map of a healthy and RIF endometrium at the WOI.

**Method:** In the current study, the endometrium from RIF patient (n = 6; age range, 32 - 35 years) and control (Ctrl) (n = 3; age range, 29 - 35 years) groups were studied at a single-cell resolution. single-cell RNA-seq and analysis were performed on the endometrium of patients with RIF and Ctrl. Immunofluorescence, flow cytometry assays, and quantitative real-time polymerase chain reaction (qRT-PCR) were performed to verify cellular identity and function.

**Results:** We profiled the transcriptomes of 60222 primary human endometrial cells isolated from control and RIF patients at a single-cell resolution. We discovered dramatic differential expression of endometrial receptivity-related genes in four major endometrial fibroblast-like cells from RIF patients compared to the control endometrium. We observed that CD49a^+^CXCR4^+^NK cells were diminished in proportion with RIF. The decrease in subset of CD63^high^PGR^high^ endometrial epithelial cells with high levels of progesterone receptor, autophagy and exosomes should contribute to the decrease in subset of NK cells. Additionally, we characterized aberrant molecular and cellular characteristics and endometrial cell-cell communication disorders in RIF patients.

**Conclusion:** Our study provides deeper insights into endometrial microenvironment disorder of RIF that are potentially applicable to improving the etiological diagnosis and therapeutics of unexplained RIF.

## Introduction

Successful implantation requires synchronized and coordinated crosstalk between the embryo and endometrium 1. In recent decades, *in vitro* fertilization-embryo transfer (IVF-ET) has become an effective treatment for infertility with improvements in laboratory procedures and ovarian stimulation. However, it is estimated that approximately 10% of women receiving IVF treatment will experience recurrent implantation failure (RIF), which refers to failure to achieve a clinical pregnancy after transfer of at least four good-quality embryos in a minimum of three fresh or frozen cycles in a woman < 40 years of age 2. Excluding structural and chromosomal abnormalities (e.g, abnormal uterine cavity, hydrosalpinx and abnormal karyotype), disorders of endometrial receptivity (i.e., progesterone resistance, shifted window of receptivity, decreased mucin 1 (MUC1) and integrin expression, and immunologic disturbances) have been considered important contributors to unexplained RIF 3, 4. However, a systematic characterization of endometrial dysfunction in unexplained RIF has not yet been fully revealed in the window of implantation (WOI).

Unlike other tissues, the human endometrium undergoes periodic variations in menstruation, menstrual repair, proliferation, and secretory differentiation, which are controlled by a sequential, and sophisticated timed interplay of female sex hormones during the menstrual cycle 5. Endometrial receptivity is defined as “the period of endometrial maturation during which the trophectoderm of the blastocyst can attach to the endometrial epithelial cells and subsequently invade the endometrial stroma and vasculature” 6. The establishment of endometrial receptivity is primarily coordinated by estrogen and progesterone, leading to dramatic functional changes in all endometrial cell types, including the stroma, glandular and luminal epithelium, resident immune cells, and endothelium 6. There is an abundance of molecular mediators involved in regulating endometrial receptivity, including adhesion molecules, cytokines, growth factors, and lipids (e.g., IGFBPs, PRL, HOXA10, WNT, and LIF) 7. During the last few decades, microarray and RNA sequencing techniques in whole-tissue transcriptomic analysis have been translated into clinical practice to evaluate the endometrial receptivity and determine the WOI timing for IVF-ET 8, 9, however, the transcriptome characterization of WOI needs further study 10, 11.

Recent studies have employed single-cell RNA sequencing (scRNA-seq) technology to investigate the cellular composition and intercellular communication events of the human endometrium during the menstrual cycle 5, 12. However, the composition changes (cell types, molecular profiles and cellular dialogue regulatory networks) of the endometrium of RIF patients during the WOI and a detailed understanding of cellular interactions in the endometrium that support endometrial receptivity and differentiation are largely unknown. Here, we profiled the endometrial cells present at the WOI timing in RIF patients and healthy controls using scRNA-seq, and provided a detailed molecular and cellular map of a healthy and RIF endometrium at the WOI.

## Materials and Methods

### Patients and Sample Collection

The protocol for this study was approved by the Human Research Ethics Committees of Obstetrics and Gynecology Hospital of Fudan University (2019-103) and Shanghai Ninth People's Hospital Affiliated Shanghai JiaoTong University School of Medicine (SH9H-2020-TK6-1). Written informed consent was obtained from all participants. Human endometrial tissues were collected from women attending the Department of Assisted Reproduction, a dedicated research clinic at the Shanghai Ninth People's Hospital Affiliated Shanghai JiaoTong University School of Medicine. Surplus tissue from endometrial biopsies obtained for diagnostic purposes at the Department of Assisted Reproduction was used for this study. For scRNA-seq, endometrial biopsy was performed five days after ovulation (ultrasonic observation, equated to LH + 7, the WOI time) in a natural cycle. The endometrium from the RIF patient group (n = 6; age range, 32 - 35 years) defined as unsuccessful implantation following transfer of at least six morphologically good-quality embryos in three or more embryo transfer cycles, was collected. The endometrium of the control (Ctrl) group (n = 3, age range, 29 - 35 years) defined as previous fertility history but with mechanical obstruction of fallopian tube or infertility due to male factors, was also obtained. Patient characteristics are shown in **Table 1**. Inclusion and exclusion criteria are listed in **Table 2**. For immunofluorescence and flow cytometry analyses, the endometrium from the RIF group (n = 12; age range, 25 - 34 years) or control (n = 58; age range, 25 - 33 years) during proliferative, secretory or menstrual phases was obtained. All donors had regular menstrual cycling (6 - 7 days every 28 - 30 days). Women with the following conditions were excluded from tissue collection: recent contraception (intrauterine device and hormonal contraceptive use in the past three months), endocrine metabolic abnormalities (i.e., polycystic ovary syndrome, diabetes, insulin resistance, and hypothyroidism), genetic abnormalities, severe adenomyosis or endometriosis, severe hydrosalpinx, moderate to severe intrauterine adhesions, uterine malformations, recurrent miscarriage, thrombosis, autoimmune diseases, and body mass index (BMI) > 30.

For *in vitro* trials, normal endometrial samples were collected from the Obstetrics and Gynecology Hospital of Fudan University, and taken from 58 patients (age range; 30 - 45 years) who underwent diagnostic curettage or hysterectomy for benign reasons (e.g., septate uterus) unrelated to endometrial dysfunction as healthy controls. These samples were evaluated by a histopathologist to identify the cyclic phase as the secretory phase and exclude endometrial pathology.

### Single-Cell Dissociation

The endometrial tissues were washed with ice-cold PBS to remove any remaining blood. The endometrial tissues were then sectioned into 1 mm^3^ pieces on ice and digested with 1 mg/mL collagenase type IV (Sigma-Aldrich, USA) for 15-20 min at 37 °C with constant agitation. After digestion, the samples were sieved through a 70 µm cell strainer (Falcon, USA), and the cell suspensions were centrifuged at 400 × g for 7 min to collect all the cells. To remove the remaining erythrocytes, 15 mL of red blood cell lysis buffer (Beijing Solarbio Science & Technology Co., Ltd., Beijing, China) was added to the pellet for 15 min on ice. After washing with PBS containing 0.04% BSA, the cell pellets were re-suspended in PBS containing 0.04% BSA and re-filtered through a 35 μm cell strainer (Falcon, USA), and the filtrate was collected. Dissociated single cells were then stained with acridine orange/propidium iodide (AO/PI) for viability assessment using a Countstar Fluorescence Cell Analyzer. The proportion of living cells was great than 90%. The single-cell suspension was further enriched with a MACS Dead Cell Removal Kit (Miltenyi Biotec, Germany).

### Single-Cell Sequencing

The scRNA-Seq libraries were generated using the 10X Genomics Chromium Controller Instrument and Chromium Single Cell 3' V3.1 Reagent Kits (10X Genomics, Pleasanton, CA, USA). Briefly, cells were concentrated to 1000 cells/µL and approximately 8,000 cells were loaded into each channel to generate single-cell gel bead-in-emulsions (GEMs), which resulted in the expected mRNA barcoding of 6000 single-cells for each sample. After the RT step, the GEMs were broken and barcoded-cDNA was purified and amplified. The amplified barcoded cDNA was fragmented, A-tailed, ligated with adaptors and amplified using PCR. The final libraries were quantified using the Qubit High Sensitivity DNA assay (Thermo Fisher Scientific, USA), and the size distribution of the libraries was determined using a High-Sensitivity DNA chip on a Bioanalyzer 2200 (Agilent). All the libraries were sequenced using an Illumina sequencer (Illumina, San Diego, CA, USA) on a 150 bp paired-end run.

### Single-Cell RNA Statistical Analysis

Single-cell RNA-seq data analysis was performed by NovelBio Bio-Pharm Technology Co., Ltd. with the NovelBrain Cloud Analysis Platform (https://singlecell.novelbrain.com/login). We applied fastp 13 with default parameters to filter the adaptor sequence and removed low-quality reads to achieve clean data. Feature-barcode matrices were then obtained by aligning reads to the human genome (GRCh38 Ensemble: version 91) using CellRanger v3.1.0. We applied the down sample analysis among samples sequenced according to the mapped barcoded reads per cell of each sample and finally achieved the aggregated matrix. Cells containing over 200 expressed genes and mitochondrial UMI rates below 40% passed the cell quality filtering and mitochondria genes were removed from the expression table.

Seurat package (version: 3.1.4, https://satijalab.org/seurat/) was used for cell normalization and regression based on the expression table according to the UMI counts of each sample and percentage of mitochondria rate to obtain scaled data. We used the cellranger aggr pipeline to combine multiple samples. The samples were down sampled according to the mapped barcoded reads per cell of each sample and the aggregated matrix was finally achieved. To remove batch effects among samples, we integrated the samples using canonical correlation analysis, which was implemented in the Seurat workflow.

PCA was constructed based on the scaled data with top 2000 high variable genes and top 10 principals were used for tSNE and UMAP constructions. CCA analysis in the Seurat package was used to correct for batch effects among the samples. Utilizing the graph-based cluster method, we acquired the unsupervised cell cluster result based on the PCA top 10 principal components. We calculated the marker genes using the FindAllMarkers function with the Wilcoxon rank sum test algorithm under following criteria: 1) lnFC > 0.25; 2) P value < 0.05; and 3) min.pct > 0.1. To identify the cell types in detail, clusters of similar cell type were selected for re-tSNE analysis, graph-based clustering and marker analysis.

### Pseudo-Time Analysis

Single-cell Trajectory analysis was performed using Monocle2 (http://cole-trapnell-lab.github.io/monocle-release) DDR-Tree and default parameters. Before Monocle analysis, we selected marker genes from the Seurat clustering results and raw expression counts of the filtered cells. Based on the pseudo-time analysis, branch expression analysis modeling (BEAM Analysis) was applied for branch fate determined gene analysis.

### Cell Communication Analysis

To enable a systematic analysis of cell-cell communication molecules, we applied cell communication analysis based on CellPhoneDB 14, a public repository of ligands, receptors, and their interactions. Membrane and secreted and peripheral proteins of these clusters were annotated. Significant mean and cell communication significance (p-value < 0.05) were calculated based on the interaction and normalized cell matrix achieved by Seurat normalization.

### QuSAGE Analysis (Gene Enrichment Analysis)

To characterize the relative activation of a given gene set such as pathway activation, “Angiogenesis” and “Fatty Acid Metabolism” as described before, we performed QuSAGE 15 (2.16.1) analysis.

### Differential Gene Expression Analysis

To identify differentially expressed genes among samples, the FindMarkers function with the Wilcoxon rank sum test algorithm was used under the following criteria: 1) lnFC > 0.25; 2) p value < 0.05; 3) min.pct > 0.1.

### Gene Ontology (GO) Functional Enrichment

Functional enrichment analysis was performed using GO enrichment analysis (http://www.geneontology.org), and each enriched ontology hierarchy (false discovery rate (FDR) < 0.05) was reported with two terms in the hierarchy: 1) the term with the highest significance value; and 2) the term with the highest specificity.

### Immunofluorescence

Paraffin sections were technically supported by Wuhan Servicebio Technology Co., Ltd. (China). Endometrial tissues fixed in 4% paraformaldehyde were embedded in paraffin and sliced to thickness of 4 μm for immunofluorescence. Endometrial tissue sections were baked at 60 °C for 2 h, deparaffinized with dimethylbenzene, and rehydrated using ethanol series. Antigen retrieval was performed by boiling the tissue sections in 10 mM Tris-EDTA buffer (pH 9.0) (Beijing Solarbio Science & Technology Co., Ltd., China) for 20 min, followed by immediate cooling in cold water for 30 min. Tissue permeabilization was performed using 0.25% Triton X-100 in PBS for 5 min, followed by washing twice with 0.05% Triton X-100 in PBS for 5 min. Nonspecific binding was blocked with 5% BSA/0.05% Triton X-100/4% goat serum in PBS for 1 h at room temperature. Tissue sections were then incubated with primary antibodies overnight at 4 °C and secondary antibodies for 1 h at room temperature. The primary antibodies and dilution ratios were as follows: CD63 (1:100; no. ab1318, Abcam, USA), CCNL2 (1:200; no. PA5-62738; Thermo Fisher Scientific), KI67 (1:200; no. ab16667, Abcam), MMP14 (1:100; no. ab3644, Abcam), PGR (1:100; no. ab63605, Abcam), and RPL10 (1:100; no. PA5-101098, Thermo Fisher, USA). The secondary antibodies used and dilution ratios were as follows: donkey anti-rabbit antibody (1:500, no. ab150075, Abcam). All the sections were counterstained with DAPI (Thermo Fisher Scientific) and mounted with buffered glycerol. Images were visualized using fluorescent signals from different lasers and captured using an optical and epifluorescence microscope (BX53 Microscope, Olympus Corporation, Japan).

### Flow Cytometry Assays

Human antibodies for flow cytometry assays (all antibodies were purchased from BioLegend, CA, USA) were used for the measurement of cell markers, as listed in **Table S1**. Isotype IgG antibody (5 μL separately) was used as control. Human Trustain FcX (BioLegend) was used to block Fc receptors prior to flow cytometry. Subsequently, the cells were washed twice and resuspended in PBS for flow cytometry. Samples were analyzed using a CytoFLEX flow cytometer (Beckman Coulter, Inc., USA) and data were analyzed using FlowJo (version 10.07 (FlowJo LLC, USA).

### Integration Analysis of the Protein-Protein Interaction (PPI) Network

The STRING database (available online: http://string-db.org) was used for PPI network prediction.

### Cells Culture Experiments

The endometrial tissues were digested and isolated as previous procedures 16, 17. After centrifugation, the supernatant of single cells was discarded, and the cells were resuspended in DMEM/F-12 containing 10% FBS (Gibco, Germany), plated in culture flasks, and incubated in a humidified incubator with 5% CO_2_ at 37 °C. Primary fibroblast-like endometrial stromal cells (ESCs) were allowed to adhere for 20 min. The culture medium was replaced every 2-3 days.

The human endometrial epithelial cell line (hEEC, WHELAB C1225) was provided by SHANGHAI WHELAB BIOSCIENCE LIMITED, and was cultured and resuspended in MEM containing 10% FBS and 1% penicillin/streptomycin. The hEECs and primary ESCs were seeded in a 24 well plate, adhered for 12 h, washed with PBS, and fixed with 4% paraformaldehyde. For characterization of hEECs, cells were then immunostained with the anti-PAX8 (1:200; no. 10336-1-AP, Proteintech, USA), anti-ER-α (1:200; no. ab32063, Abcam, USA), anti-EpCAM (1:800; no. 2929S, CST, USA) and anti-CK7 (1:100; no. ab185048, Abcam, USA) antibodies overnight at 4 °C and secondary antibodies (donkey anti-rabbit antibody (1:500, no. ab150075, Abcam) or donkey anti-mouse antibody (1:500, no. ab150105, Abcam) for 1 h at room temperature. Cells were then counter stained with 1 µg/mL DAPI for 10 min for nuclear labling, and visualized by a fluorescence microscope (BX53 Microscope, Olympus Corporation, Japan). Images were processed using ImageJ (National Institutes of Health, USA).

The hEECs were treated with the vehicle (0.1% DMSO, Sigma, USA) or medroxyprogesterone acetate (MPA) (1 μM, Sigma USA) for 24 h, *in vitro*. The cell culture supernatant was collected to extract exosomes.

The hEECs were treated with rh-IGF1 (2 ng/mL, Abcam, USA) for 48 h, and collected to determine the mRNA expression levels of *ATG5*, *ATG7*, *BECN2*, *MAP1LC3B*, *mTOR*, and *MUC1* using quantitative real-time polymerase chain reaction (qRT-PCR).

Additionally, primary ESCs were treated with vehicle or palmitic acid (PA) (10 μM, Xi'an Kunchuang Co., Ltd., China) for 48h, *in vitro*. The hEECs were also treated with the vehicle or PA (10 μM) for 48h, or GW4869 (1 μM, MedChem Express, USA) for 24h, *in vitro*. These cells were then collected, and the expression of APOD, APOE, IL15 and CXCL12 was detected by qRT-PCR.

### Isolation and Purification of Endometrial NK cells

The endometrium tissues were digested and isolated as a previous procedure 16, 17. Single cells were collected to isolate endometrial NK cells by MASC, a human NK cell isolation kit (130-092-657, Miltenyi Biotec, Germany) for *in vitro* experiments. NK cells were co-cultured with EECs pre-treated with GW4869 or the vehicle (0.1% DMSO, USA) for 24h, and then NK cells were collected and further analyzed by flow cytometry assays.

### Exosomes Enrichment, Characterization, Purification and Analysis

Details can be found in Supplementary information.

### Quantitative Real-Time Polymerase Chain Reaction

Total RNA from primary ESCs and hEECs was extracted by TRIzol regent (Invitrogen, Carlsbad, CA, USA). Subsequently, a NanoDrop spectrophotometer (NanoDrop Technologies; Thermo Fisher Scientific, MA, USA) was used to quantify the concentration and purity of RNA. The PrimeScript RT Reagent Kit (TaKaRa Biotechnology, Co., Ltd., Dalian, China) was used to hereversely transcribe total RNA to cDNA. Next, qRT-qPCR was performed using the SYBR Green PCR Master Mix (TaKaRa Biotechnology). The qRT-PCR primers used are listed in **Table S2**. The target mRNA expressions were normalized to *ACTB* expression. All reactions were performed using an Applied Biosystems 7500 Real-Time PCR System (Thermo Fisher Scientific). The test results were analyzed using the 2^-ΔΔ^Ct method.

### Statistics

The continuous variable is shown as mean ± SEM for normally distributed data, and as median ± inter-quartile range (IQR) for non-normally distributed data. Continuous variables were analyzed using Student's *t*-test for normally distributed data or two-tailed Mann Whitney test for non-normally distributed data. All analyses were performed using SPSS 21.0 Statistical Package. P < 0.05 was considered to indicate a statistically significance.

## Results

### Atlas of the Endometrium of RIF Patients

**Figure 1:**
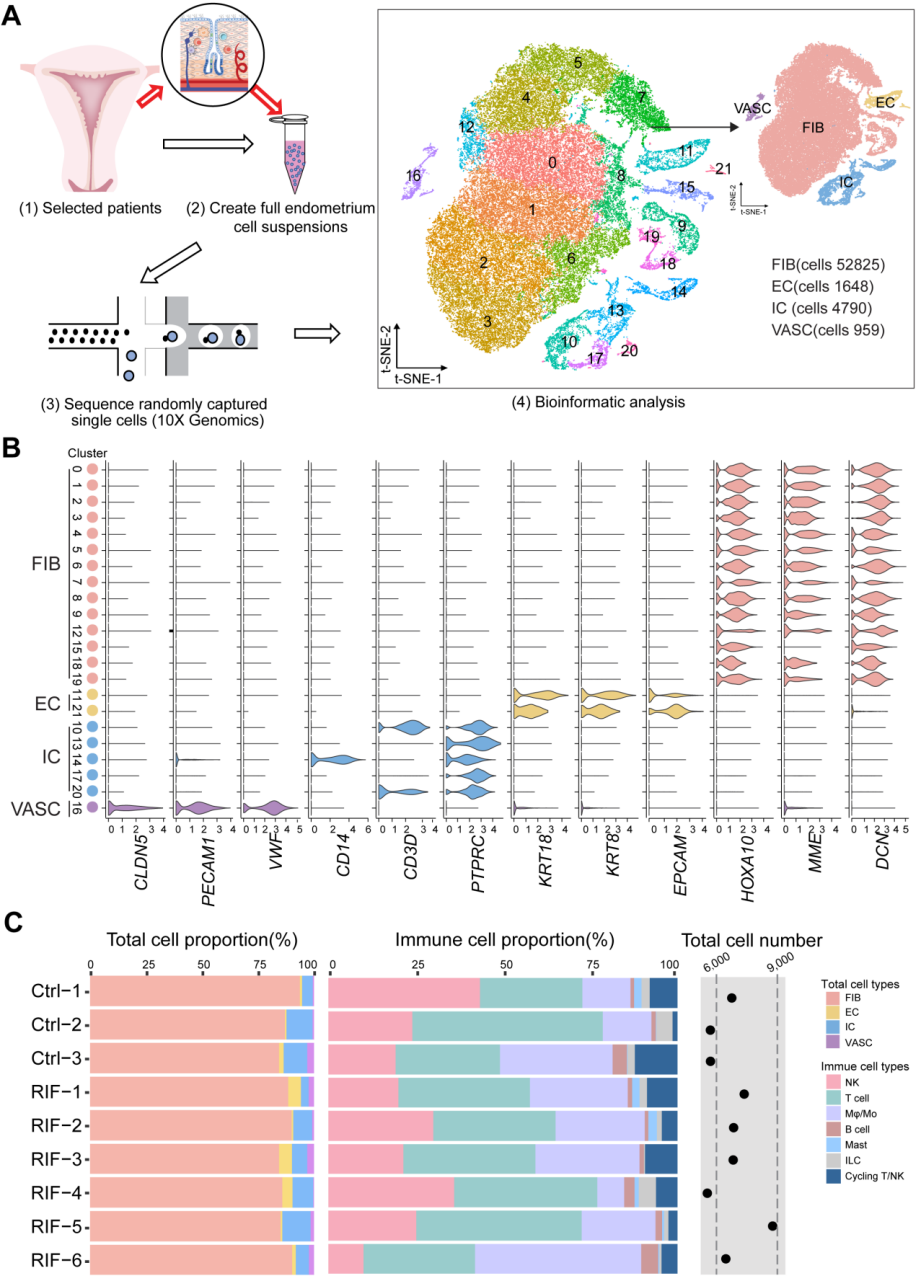
** Atlas of the endometrium of healthy controls and RIF patients. (A)** Flowchart overview of the scRNA-seq and methodology (left), and t-SNE plots on 60222 single cells in the endometrium of three control and six RIF patients (right), indicating 22 clusters and four major cell types (FIB, EC, IC and VASC). **(B)** Violin plots of representative marker genes for 22 clusters and 4 cell subsets. The X-axis is a log scale normalized read count. **(C)** Proportion and total cell number of FIB, VASC, IC and the subpopulation of IC (NK, T, Mφ/Mo, B, Mast, ILC and T/NKp cells) in the endometrium of different samples. FIB: fibroblast-like cells; EC: epithelial cells; IC: immune cells; VASC: vascular cells.

To determine the full repertoire of cell types and gene expression programs present in the endometrium, we isolated cells from the endometrium of patients with RIF (n = 6) and healthy controls (n = 3) at the time of WOI (see the Materials and Methods section), and generated single-cell transcriptome libraries on the droplet-based 10X Genomics Chromium System (**Figure 1A**). After computational quality control and integration of transcriptomes, we obtained a total of 60222 endometrial single-cell transcriptomes, preformed graph-based clustering of *t*-disturbed stochastic neighbor embedding (*t*-SNE) and used cluster-specific marker genes to annotate the clusters (**Figure 1A** and **Figure S1**). Overall, all sequenced endometrial cells were assigned to four main classes: fibroblast-like cells (FIB, expressing *HOXA10*, *MME* and *DCN*; 52825 cells), epithelial cells (EC, expressing *KRT18*, *KRT8* and *EPCAM*; 1648 cells), immune cells (IC, expressing *PTPRC*; 4790 cells) and vascular cells (VASC, expressing *CLDN5*, *PECAM1*, and *VWF*; 959 cells) (**Figure 1A**-**B**).

Although the exact mechanism is largely unknown, there is evidence of a large amount of immune cell infiltration in the human endometrium at the WOI, including NK cells, macrophages and T cells 18. Here, we also observed a very rich population of immune cells, such as NK cells (*PTPRC*^+^*CD3E*^-^*NACM1*^+^*NKG7*^+^), monocyte/macrophage (Mo/Mφ, *PTPRC*^+^*CD14*^+^*TYROBP*^+^), CD4^+^T cells (*PTPRC*^+^*CD3E*^-^*CD4*^+^*CD8A*^-^), CD8^+^T cells (*PTPRC*^+^*CD3E*^-^*CD4*^-^*CD8A*^+^), B cells (*PTPRC*^+^*CD3E*^-^*CD19*^+^*CD79A*^+^), mast cells (*PTPRC*^+^*HPGD*^+^), ILC (*PTPRC*^+^*IL4I1*^+^), and T/NKp cells (*PTPRC*^+^*CD3E*^+^*NACM1*^+^*NKG7*^+^*MKI67*^+^) (**Figure S2A**-**C**, **Figure S3**). Here, digestive conditions and filtration operations may limit EC cell acquisition. Notably, we observed no difference in the total cell number and proportion of FIB, EC, VASC, IC and the subpopulation of IC (NK, T, Mo /Mφ, B, Mast, ILC and T/NKp cells) in the endometrium between the Ctrl and RIF patients (**Figure 1C**).

### Identification of Population of Human Endometrial Fibroblast-like Cells at the WOI

**Figure 2:**
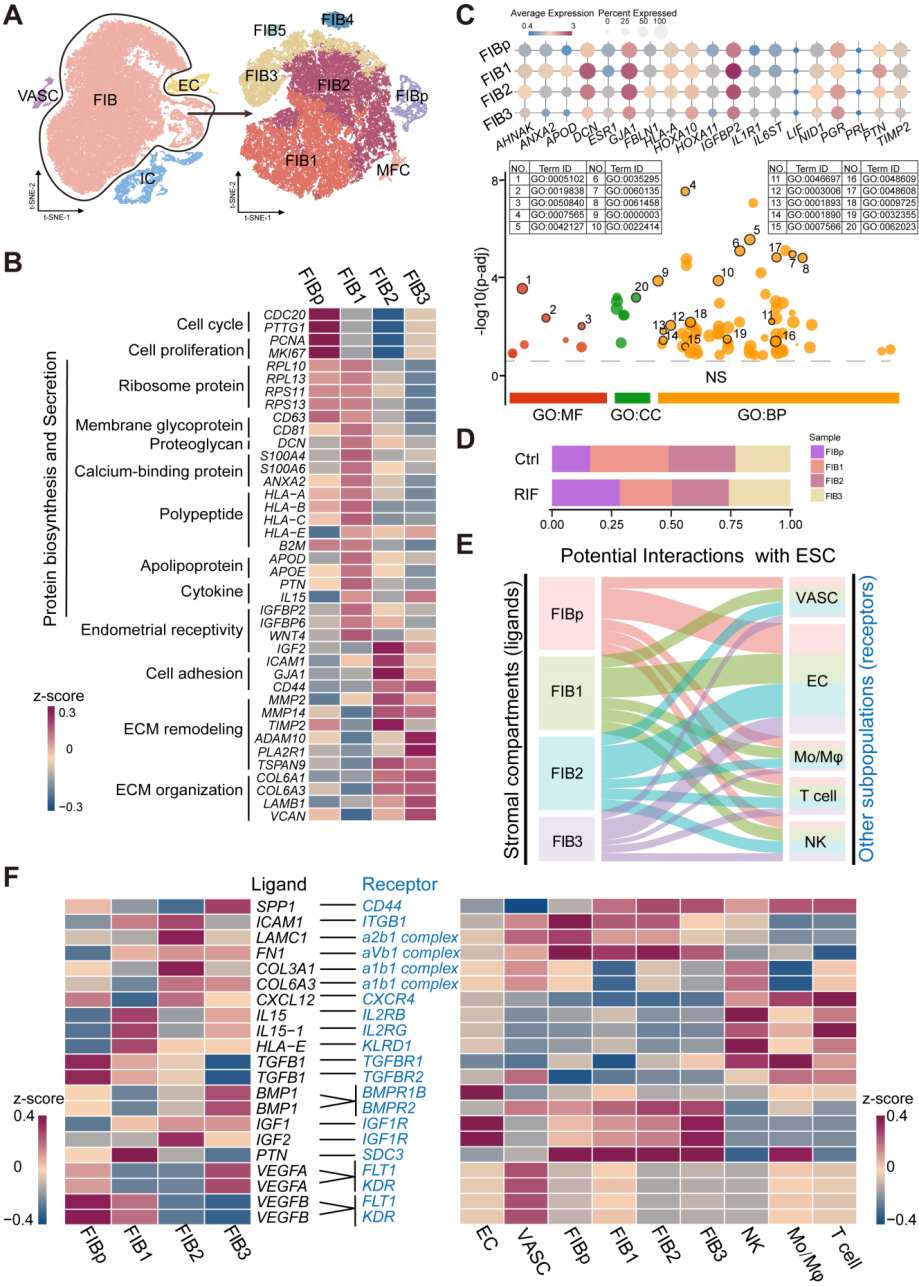
** Identification of population of human endometrial FIB at the WOI. (A)** Cell cluster of FIB (52825 cells) was re-clustered into seven sub-clusters visualized by t-SNE. **(B)** Heat map showing relative expression (z-score) of selected genes for four main sub-clusters of endometrial FIB. **(C)** Bubble diagram showing average expression of endometrial receptivity-related genes (such as *HOXA10*, *IGFBP2*,* LIF*, and *PRL*) for four FIBs (FIBp, FIB1, FIB2 and FIB3) (up). Manhattan plot indicating the Gene Ontology (GO) enrichment of endometrial receptivity-related genes, and the most important GO terms (the details of GO terms are showed in Figure S6) of endometrial receptivity-related genes (down). One dot represented one GO term, and the size of the dots represented the number of genes that were enriched. The Manhattan plot was drawn by an online tool - g:Profile (https://biit.cs.ut.ee/gprofiler/). **(D)** Proportions of the four FIBs between the Ctrl and RIF groups. **(E)** Potential interactions between four FIBs and other subtypes of endometrium cells based on receptor-ligand pairs analysis. Width of the line represented the number of receptor-ligand pairs. **(F)** Heat map showing selected significant ligand-receptor interactions (P value < 0.05, permutation test, see Methods) between four FIBs (left) and endometrium cells (right). Assays were carried out at the mRNA level, but are extrapolated as protein interactions.

The three main tissue compartments of the uterus support and regulate pregnancy, including the stroma, endometrial epithelium, and myometrium. As FIB is the most abundant cell type in the endometrium, we initially explored endometrial FIBs, and identified seven subset clusters of endometrial FIBs: *TOP2A*^+^*MKI67*^+^*CDC20*^+^ endometrial fibroblast-like cells (FIBp), *RPL10*^+^*RPS10*^+^*PTN*^+^*IGFBP2*^+^ FIB with high levels of protein synthesis and secretion-related genes (FIB1), *MMP14*^+^*IGF2*^+^*COL6A1*^+^*IGFBP2*^+^ FIB with high levels of tissue remodeling-related genes (FIB2), *ACTA2*^+^*RGS5*^+^ myofibroblasts (MFC), and groups of *RPL10*^low^*CD74*^+^*IL32*^high^ FIB5, *CCNL2*^+^*MMP14*^+^*TIMP2*^low^ FIB3 and *MT1G*^+^*TM4SF1*^+^ FIB4 (**Figure 2A**, **Figure S4A**-**B**). However, further analysis showed that FIB4 and FIB5 existed only in a single tissue sample as small subsets (**Figure S4C**). Considering the key role of FIB in embryo implantation 19, our subsequent analysis focused primarily on four groups of FIBs (FIBp, FIB1, FIB2 and FIB3).

Among these, FIBp had high levels of cell cycle and proliferation-related genes (e.g., *CDC20*, *PTTG1*, *PCNA* and *MKI67*), suggesting a high proliferative ability (**Figure 2B** and **Figure S5**). Additionally, this subset of FIBs expressed a certain level of ribosomal protein-related genes (e.g., *RPL10*, and *RPS11*) and HLA class I histocompatibility antigens (e.g., *HLA-A*, *HLA-B*,* HLA-C*). Maternal HLA-C has been reported to inhibit the cytotoxicity of maternal NK cells, thereby establishing maternal-fetal immune tolerance 20, 21. Notably, FIB1 had strong protein synthesis and secretion capacities, and high levels of endometrial receptivity-related molecules (**Figure 2B**-**C**, **Figure S5**, and **Figure S6A**), which were characterized by high levels of ribosome protein (e.g., *RPL10*, *RPS11*), membrane glycoprotein (e.g., *CD81*), proteoglycan (*DCN*), calcium-binding proteins (*S100A4*, *S100A6*, and *ANAX2*), HLAs, apolipoprotein (*APOD*, and* APOE*), cytokine (*PTN*, and *IL15*) and insulin like growth factor binding protein (IGFBP)-coded genes. Additionally, FIB2 highly expressed cell adhesion (e.g.,* ICAM*, *GJA1*, and* CD44*) and extracellular matrix (ECM) remodeling (e.g., *MMP2*, and *MMP14*)-related genes, as well as *IGF2* and *IGFBP*. Several adhesion-promoting molecules, such as *CD44* 22, support the presence of cross-talk between blastocysts and the endometrial epithelium/stroma during human embryonic implantation. The ECM remodeling is essential for successful implantation and placentation and multiple MMPs (MMP14 and ADAM10) and their substrates are involved in this process 23. Therefore, FIB2 had strong adhesion and tissue remodeling capacities, and high endometrial receptivity (**Figure 2B**-**C**, **Figure S5**, and **Figure S6A**). In contrast to decidualized stromal cells 14, some classical marker genes (e.g., *PRL*) of endometrial receptivity were expressed in all endometrial FIB at low levels (**Figure 2C**), which partly echoed the previous report 5. Compared with FIB2, the genes for cell adhesion, MMPs and endometrial receptivity in FIB3 were decreased (**Figure 2B**-**C**, **Figure S5**, and **Figure S6A**). Contrastingly, FIB3 highly expressed ECM organization-related genes, as well as a certain level of cell cycle and proliferation molecules. Further analysis showed decreased percentages of FIB1 and ratio of FIB2 to FIB3, and increased FIBp in RIF patients, based on the average of samples (**Figure 2D,** and **Figure S6B**).

Based on receptor-ligand pairs, potential interactions between these four clusters of FIBs and other cells (EC, VASC, NK, macrophage and T cells) in the endometrium were predicted (**Figure 2E**). Particularly, IL15 and HLA-E derived FIB1 were predicted to promote the proliferation and decrease the cytotoxicity of endometrial NK cells; the interaction of ICAM1/ITGB1, LAMC1/a2b1 complex, FN1/aVb1 complex, and COL3A1/COL6A3/a1b1 complex possibly contributed to the cell adhesion between FIB2 and other FIBs or NK cells. The *IGF2* and *BMP1* expressed by FIB2 and FIB3 were involved in the differentiation regulation of EC and FIB, which was required for endometrial receptivity and implantation 24; notably, VEGFA and VEGFB produced by FIB3 and FIBp resulted in angiogenesis of the endometrium by binding to receptors (**Figure 2F**).

### Subsets of FIBs with Poor Endometrial Receptivity and Immune Regulation are observed in RIF Patients

Previous reports have shown that the gene expression profile of total endometrial tissues displays high cellular proliferation, DNA synthesis, angiogenesis and vasculogenesis during the proliferative phase of the menstrual cycle 25. Subsequently, cell proliferation is inhibited, but the transformation and differentiation of the endometrium begins to occur, and the gene expression of metabolism, cell differentiation and communication, innate immune response, adhesion, and ECM degradation is up-regulated during the secretory phase, as well as in the glandular section 26.

**Figure 3:**
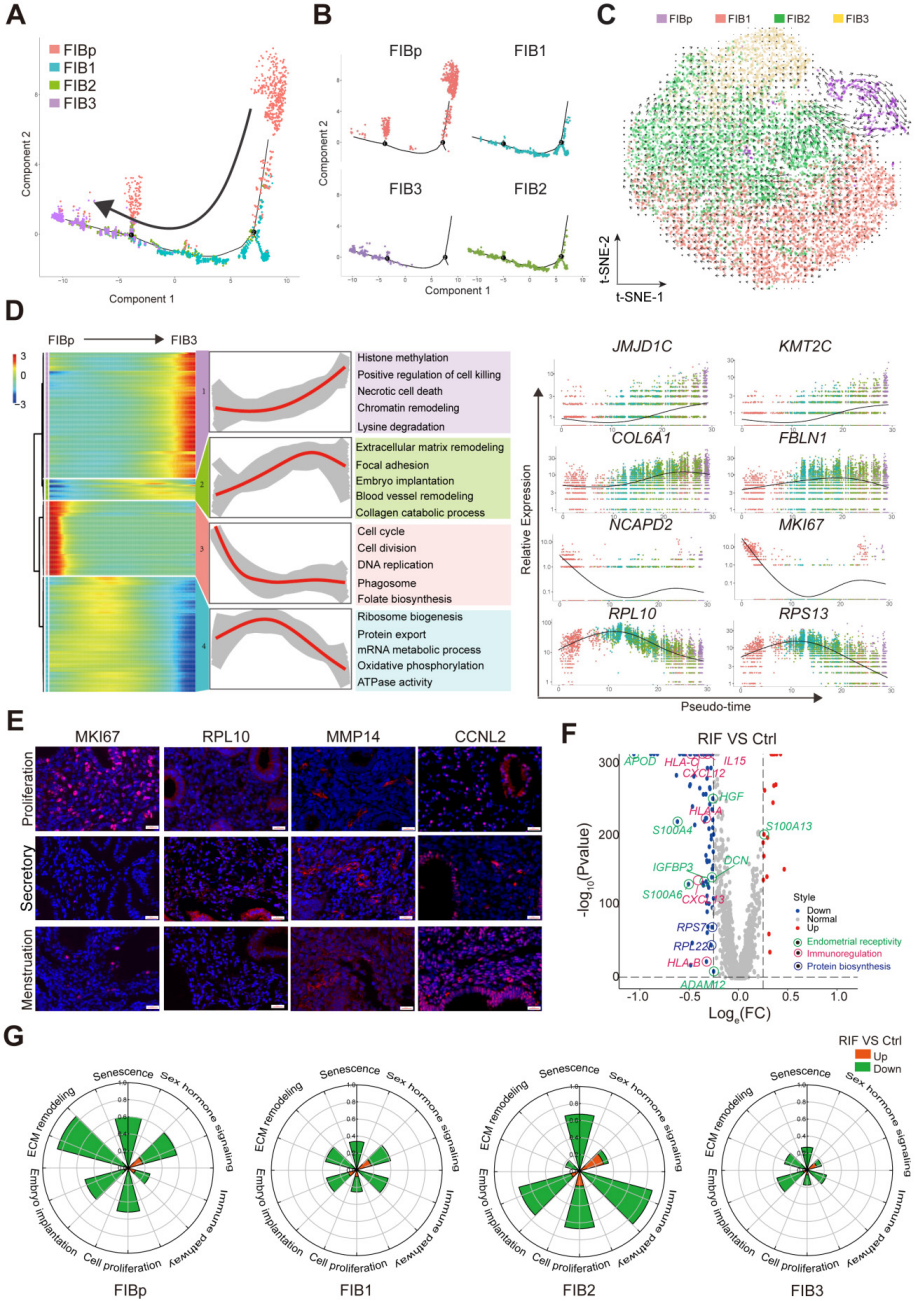
** Subsets of FIB with poor endometrial receptivity and immune regulation are observed in RIF patients. (A, B)** Monocle pseudotime trajectory showing the progression of FIBp, FIB1, FIB2, and FIB3. **(C)** t-SNE with RNA velocity vectors for FIBp, FIB1, FIB2 and FIB3. **(D)** Expression of the genes in a branch-dependent manner. Each row indicated the standardized kinetic curves of a gene. From the left to the right of the heatmap, the kinetic curve progresses from the FIBp along the trajectory to the FIB3 (left). Based on their characteristic expression dynamics, 21480 genes were categorized into 4 clusters, and the enriched GO terms for each gene cluster were identified (middle). Expression patterns of representative genes along the reprogramming trajectory (right). **(E)** Immunofluorescence staining of MKi67, RPL10, MMP14, and CCNL2 in in three different menstrual cycles of endometrial stroma from controls (n = 6 for each group). Scale bar, 20 µm (times of endometrial biopsy are showed in Table S4). **(F)** Volcano plot showing differentially expressed genes of the four FIBs between the Ctrl and RIF groups. Red color indicates genes that are upregulated and blue color indicates downregulated genes in the RIF groups compared with the Ctrl groups. **(G)** Rose diagrams indicating the proportion of upregulated (red) and downregulated (green) genes in four FIBs from the RIF groups compared with the Ctrl groups. List of genes screened is shown in Tables S2.

To further explore the relationship between the cell transformation and differentiation of these four FIBs, a standard pseudotime analysis was performed, and a new trajectory for FIBp, FIB1, FIB2 and FIB3 was constructed (**Figure 3A**). Notably, we observed a notable discontinuity among the four groups of FIBs. As shown, FIBp was the starting point, which went through FIB1 and FIB2, and the final endpoint was FIB3 (**Figure 3A**-**B**). Additionally, *t*-SNE with RNA velocity also demonstrated the evolution from FIBp to FIB3 (**Figure 3C**). The expression of cell cycle, division and DNA replication genes (e.g., *NCAPD2*, *MKI67*, and *CDC20*) was markedly reduced from the starting point (FIBp) to the other FIBs branches in the trajectory (**Figure 3D**). Gene expression (e.g, *RPL10*, *RPS13*, and* SLC25A6*) in ribosome biogenesis, protein export, and mRNA metabolic process was up-regulated early in FIB1 differentiation and down-regulated in cell differentiating into both FIB2 and FIB3 (**Figure 3D**). Importantly, gene expression (e.g, *COL6A1*, *FBLN1*, and *FN1*) in focal adhesion, embryo implantation, ECM remodeling, blood vessel remodeling peaked during FIB2 differentiation, and a decreasing trend was then observed in the FIB3 differentiation, which was characterized by high expression of histone methylation, positive regulation of cell killing, necrotic cell death, chromatin remodeling and lysine degradation-related genes (e.g., *JMJD1C*, *KMT2C*, *ELN*, and* REV3L*) (**Figure 3D**, **Figure S7**).

To confirm the single-cell trajectory, the expression of KI67, PRL10, MMP14 and CCNL12 was detected by immunofluorescence staining. As expected, *MKI67*^+^ FIBp was mainly localized in the endometrium during the proliferative phase of menstruation. During the secretory phase, *PRL10*^+^ FIB1 and *MMP14*^+^ FIB2 were enriched in the endometrium, while *CCNL2*^+^*MMP14*^+^ FIB3 rapidly accumulated during the menstrual period (**Figure 3E**). These data suggest that enrichment of FIB1 and FIB2 with high protein biogenesis, tissue remodeling and good endometrial receptivity properties at the WOI is very important for embryo implantation. Further analysis of the volcano plot showed that the gene expression of endometrial receptivity (e.g, *IGFBP3*, *S110A3*, *APOD*, and* DCN*), immunoregulation (e.g, *CXCL12*, *IL15*, and* HLA-C*) and protein biosynthesis (e.g., *RPL22*, and* RPS27*) was markedly decreased in these four FIBs from RIF patients (**Figure 3F**). Particularly, the rose diagram showed the genes in the pathways of cell proliferation, embryo implantation, ECM remodeling, senescence, sex hormone signaling and immune pathway was significantly down-regulated in these four FIBs from RIF patients (**Figure 3G**, **Table S1**). Therefore, the results suggest that the decreased levels of endometrial receptivity and immune regulation-related genes mainly expressed by FIB1 and FIB2 may lead to poor endometrial receptivity in RIF patients.

### Subset of Endometrial Epithelial Cell, EC1, is diminished in the Endometrium of RIF Patients

**Figure 4:**
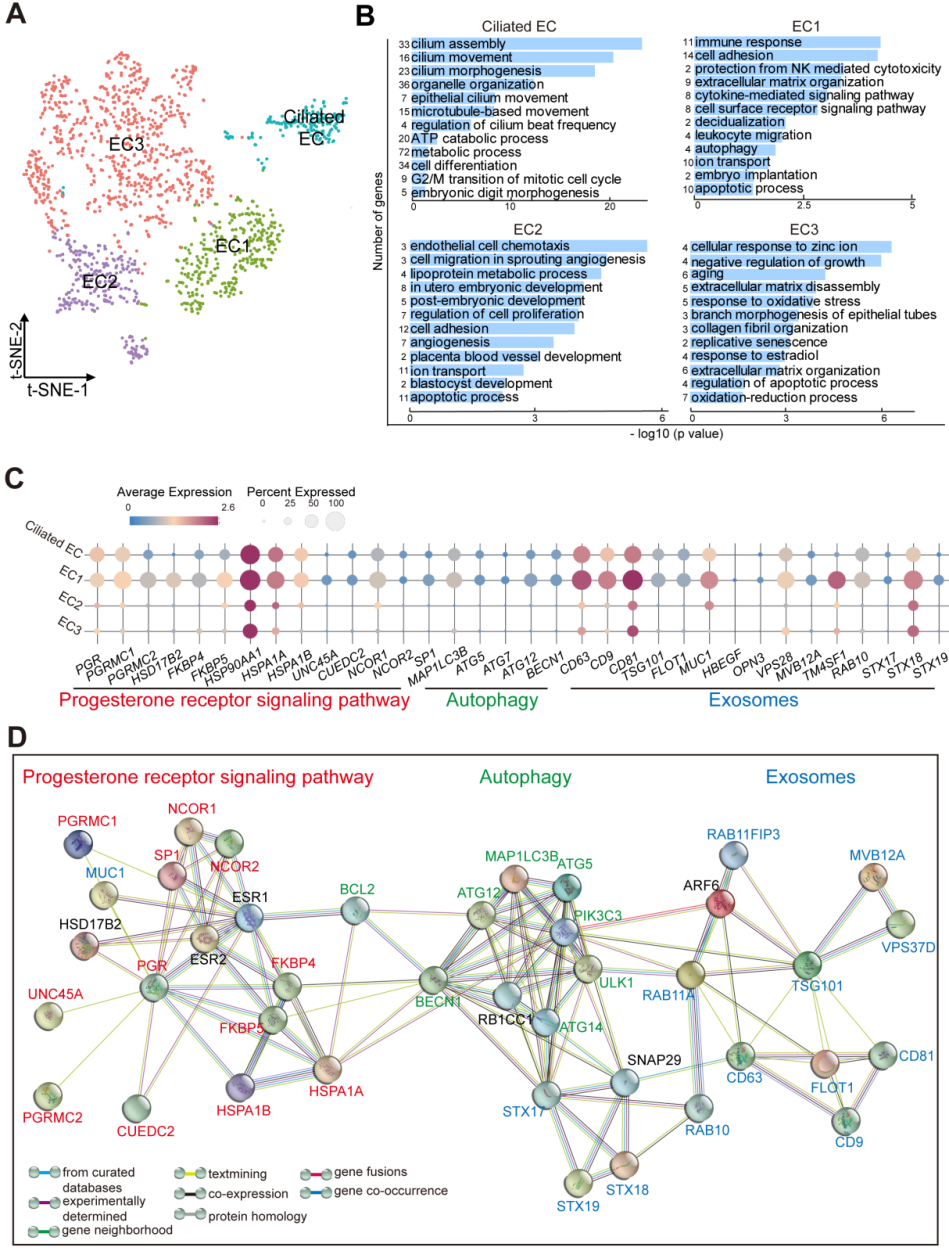
** Subset of endometrial epithelial cell, EC1, is diminished in the endometrium of RIF patients. (A)** t-SNE map of four sub-clusters (Ciliated-EC, EC1, EC2, and EC3) of EC. **(B)** Significant gene markers (listed in the Supplemental Information) for each cluster in the EC were selected to perform GO analysis. The GO terms with P < 0.05 are shown. Gene number of each GO term is listed on the left. P value is shown as -log 10 (P value). **(C)** Bubble diagram showing average expression of selected genes (progesterone receptor signaling pathway, autophagy and exosomes-related genes) for the four sub-clusters of EC. **(D)** The PPI network of selected genes in (C). Different edges color presented different interactions.

As the site of blastocyst adhesion, the epithelium is perceived as a crucial site for uterine receptivity, which transmits signals to other compartments 25. Markers that distinguished the different endometrial epithelial cell populations identified four clusters (referred to as Ciliated EC, EC1, EC2 and EC3): *FOXJ1*^+^*IGFBP7*^+^ Ciliated endometrial epithelial cell (Ciliated EC, as reported recently 5), *ALCAM*^+^*CD63*^+^*ESR1*^+^*PRDX6*^+^ EC1, *ESR1*^+^*DUOXA1*^+^*PRDX6*^-^*LDHB*^-^ EC2, and *ALCAM*^-^*CD63*^-^*IGF1*^+^*PRDX6*^+^*LDHB*^+^ EC3 (**Figure 4A**, **Figure S8A**). Functional enrichment analysis, *FOXJ1*^+^ Ciliated EC highly expressed genes involved in epithelial and ciliated cell development, cilium movement and beat frequency (**Figure 4B**). More importantly, genes involved in multiple biological pathways (e.g., cell adhesion, immune response and leukocyte migration, ECM organization, cytokine-mediated signaling pathway, decidualization, autophagy and embryo implantation) were enriched in EC1, possibly regulating endometrial receptivity during implantation (**Figure 4B**, **Figure S8B**). Genes associated with angiogenesis, blastocyst and embryo development, and cellular adhesion, were enriched in EC2. Contrastingly, the most pronounced functional feature of EC3 was a high proportion of cell senescence, replicative senescence and response to oxidative stress (**Figure 4B**).

Interestingly, EC1 displayed an activated progesterone receptor signaling (e.g, high levels of *PGR*, *PGRMC1*/*PGRMC2*, *FKBP4*/*FKBP5*, *HSPA1A*/*HSPA1B*, and *NCOR1*/*NCOR2*), contributing to the strong exosome production, transport and secretion capacities (*CD63*^+^*CD9*^+^*CD81*^+^*TSG101*^+^*MUC1*^+^*VPS28*^+^*TM4SF1*^+^*STX18*^+^) possibly by the up-regulation of cell autophagy (*MAP1LC3B*^+^*ATG5*^+^*ATG12*^+^) (**Figure 4C**-**D**). In contrast, other epithelial cells were less capable of secretion properties, especially EC2 and EC3.

**Figure 5:**
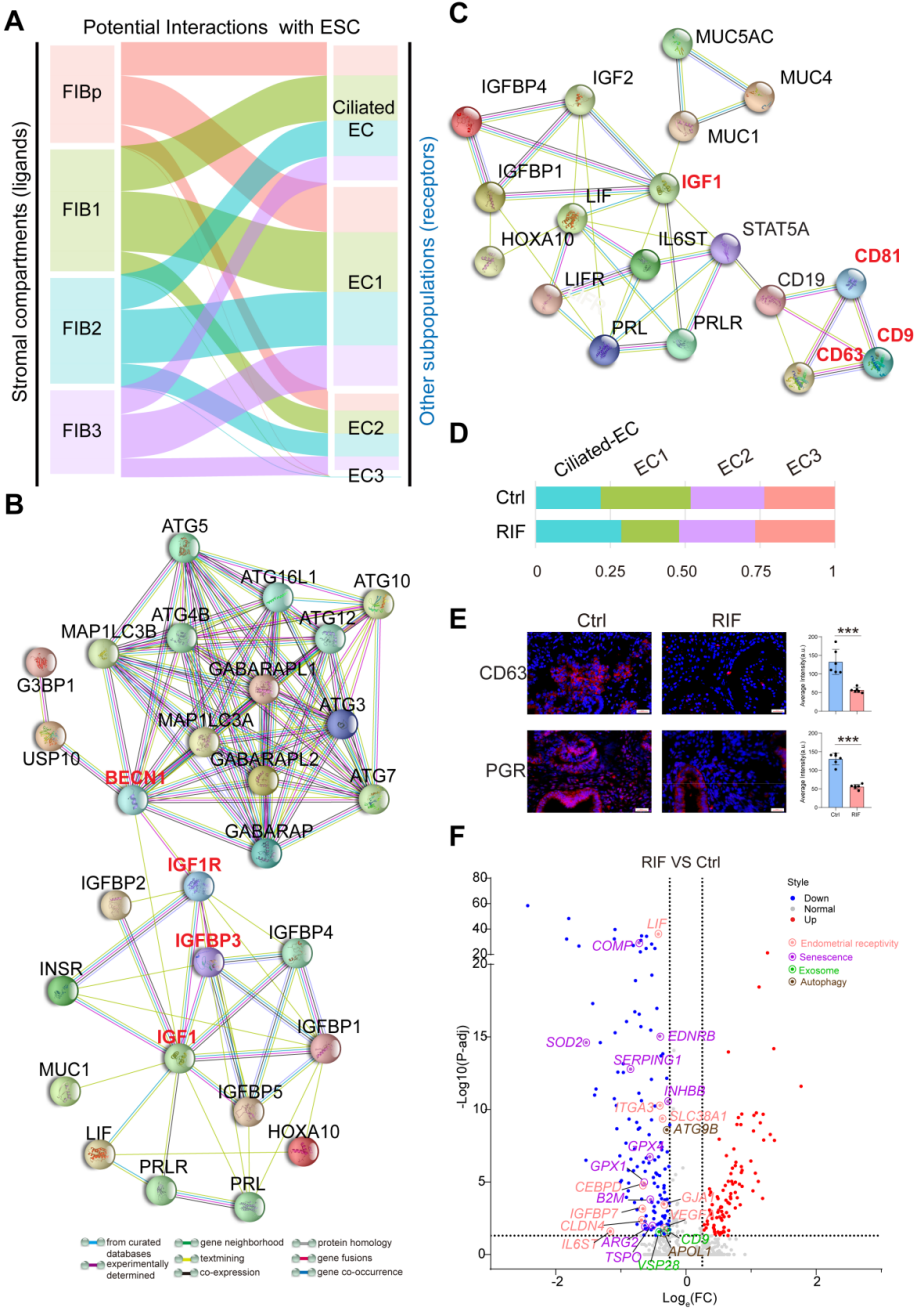
** A close interactive dialogue between endometrial stromal cells and epithelial cells. (A)** Potential interactions between four FIB (FIBp, FIB1, FIB2, and FIB3) and four EC (Ciliated-EC, EC1, EC2 and EC3) subpopulations based on receptor-ligand pairs. The width of the line represented the number of receptor-ligand pairs. **(B)** Protein-protein interaction (PPI) network of IGF, endometrial receptivity-related genes and autophagy-related genes. Different edges color presented different interactions. **(C)** Protein-protein interaction network of IGF and endometrial receptivity-related genes and exosome-related gene. Different edges color presented different interactions. **(D)** Proportion of the four sub-clusters of EC in the Ctrl and RIF groups. **(E)** Expressions of CD63 and PGR in endometrial stroma from controls and RIF patients at the WOI time were analyzed by immunofluorescence staining (n = 6 for each group). Data were presented as mean ± SEM and analyzed by t test (***, p < 0.001). Scale bar, 20 µm (times of endometrial biopsy were showed in Table S5). **(F)** Volcano plot showing differentially expressed genes of ECs between the Ctrl and RIF groups. Red color indicates genes that are upregulated and blue color indicates downregulated genes in the RIF groups when compared with the Ctrl groups.

Notably, a close interactive dialogue between endometrial stromal cells and epithelial cells was observed, particularly in the four populations of FIBs and EC1 (**Figure 5A**). To explore the potential functions of EC cells, we employed the hEEC cell line. We have verified by *in vitro* experiments that hEECs express markers characteristic of epithelial cells 27 (**Figure S9**). Further analysis showed that activated autophagy of EC1 may be induced by IGF-1, which is produced by FIB2 and FIB3, in a mTOR-independent manner (**Figure 2F**, **Figure 5B**, and **Figure S10A**). Further, MUC 1 (a highly glycosylated polymorphic mucin-like protein) is more abundant in fertile women than in infertile women, and has also been shown to be progesterone- rather than estrogen-dependent in baboons, serving as a marker of the pre-implantation phase 28. Here, we observed that *MUC1*^+^ EC1 with many exosomes had better endometrial receptivity (expressed high levels of *IGFBP2*, *IL-1*, and *IL6*; **Figure S8**), possibly by interacting with IGF1 signaling (**Figure 5C**). Stimulation with MPA led to increased exosome production, *in vitro* (**Figure S10B**-**C**). Extracellular vesicles (EVs) were recently shown to play a role in embryo-mother cross communication, even at preconception from gamete maturation to implantation and throughout pregnancy 29, 30. However, further analysis and verification showed that the percentage of *CD63*^+^EC1 with high response to progesterone was decreased significantly in RIF patients (**Figure 5D**-**E**). Moreover, many candidate genes of endometrial receptivity (such as *LIF*, *IL6ST*, and *ITGA3*), senescence (such as *COMP*, *SOD2*, and *EDNRB*), exosome (such as *CD9*, and *VSP28*) and autophagy (such as *ATG9B*, and* APOL1*) were downregulated in ECs of RIF patients (**Figure 5F**).

### Endometrial *CD49a*^+^*CXCR4*^+^ NK2 Cell is Decreased in RIF Patients

**Figure 6:**
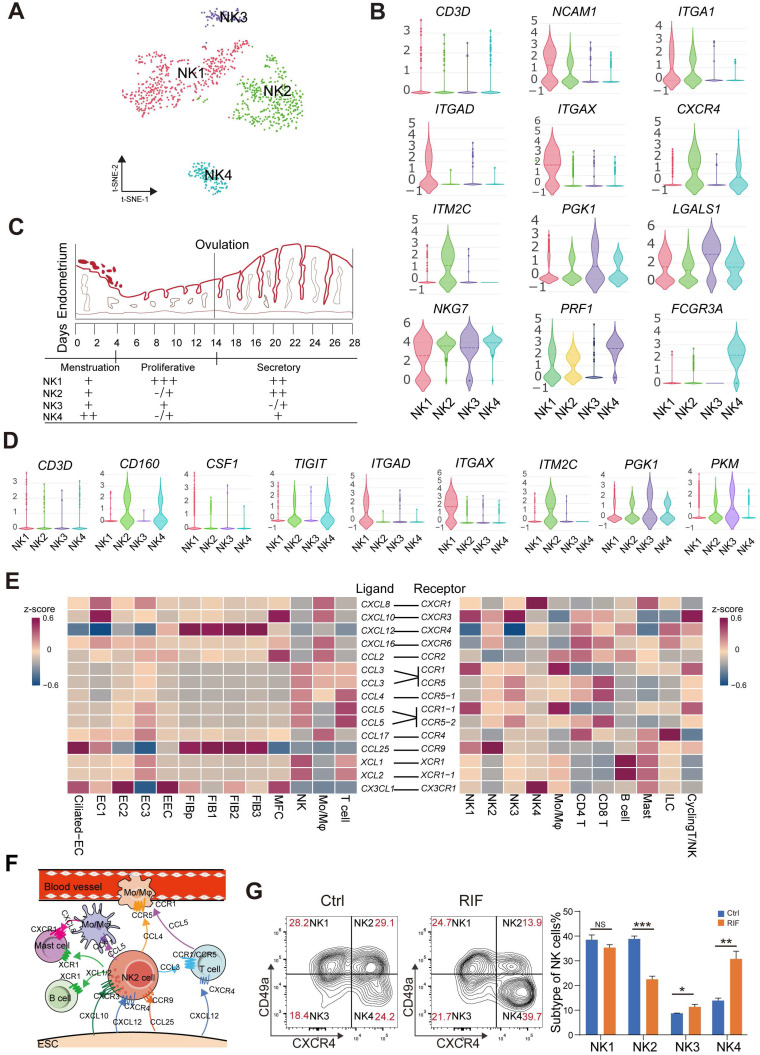
** Endometrial *CD49a*^+^*CXCR4*^+^ NK2 cells are decreased in the RIF patients. (A)** t-SNE map of four sub-clusters of endometrial NK cells. **(B)** Expression of representative marker genes in Violin plots. **(C)** Changes of NK1, NK2, NK3, and NK4 cellular proportion in total endometrial CD45^+^CD3^-^CD56^+^ NK cells across the menstrual cycles were analyzed using FCM analysis (-/+: < 10%, +: < 20%, ++: < 50%, +++: ≥ 50%). **(D)** Expression of representative marker genes in the Violin plots. **(E)** Heat map of CellPhoneDB showing selected significant ligand-receptor interactions (P value < 0.05, permutation test, see Methods) between all subtypes of endometrium cells (left) and all immunocytes (right). Assays were carried out at the mRNA level, but are extrapolated as protein interactions. **(F)** Diagram of the main chemokine and receptors expressed on the FIBs and immunocytes that are involved in cell migration and recruitment. **(G)** Proportions of the four NK cellular subtypes in the endometrial stroma in the controls and RIF patients (n = 6, for each group) were analyzed by flow cytometry. Cells were initially gated within CD45^+^CD3^-^CD56^+^ gate, and then CXCR4 and CD49a gating. Data were presented as mean ± SEM and analyzed by t test (NS, no significance, *, p < 0.05, **, p < 0.01, ***, p < 0.001).

Endometrial NK cells play an important role in the decidualization, angiogenesis and embryo implantation 31, 32. We then identified four main NK cellular subsets (NK1, NK2, NK3 and NK4) (**Figure 6A**). The NK1 cells expressed *ITGA1* (also known as *CD49a*, a tissue-resident marker), *CD103* and *ITGB2*, but not *CXCR4* (an important chemokine receptor for NK cell migration and recruitment), *CD39*, *KIR2DL1*, *CD160*, or *FCGR3A* (also known as *CD16*) (**Figure 6B**, and** Figure 6D**). This subset of resident NK cells should be involved in tissue remolding by ECM-receptor interaction (**Figure S11A**), and may be further differentiated to *CD103*^-^*ITGB2*^+^*CD39*^-^*KIR2DL1*^-^ decidual NK cells (dNK2), as reported in a previous study 14. The NK2 cells expressed *CD49a*, *CXCR4*, *CD103*, *ITGB2*, *CD160* and *TIGIT*, but not *CD39*, *KIR2DL1* and *CD16* (**Figure 6B**, and **Figure 6D**), which might be the precursor cells of *CD49a*^+^*CD103*^+^*ITGB1*^+^*KIR2DL1*^-^ dNK3 in the decidua during early pregnancy 14. Contrastingly, NK3 cells highly expressed *LGALS1* and *PGK1*, and negatively expressed *CD49a*, *CXCR4*, *CD39*, *ITGB2*, *KIR2DL1*, *PRF1* and *CD16* (**Figure 6B**, and** Figure 6D**). *CD49a*^-^*CXCR4*^+^*CD160*^+^*TIGIT*^+^*PRF1*^+^*CD16*^+^ NK4 cells displayed an activated cytotoxicity. Unlike peripheral blood NK cells, the NK4 cells exhibit high levels of *ITGB2*. Interestingly, we found that the percentage of the four subsets of human endometrial NK cells also showed dynamic and periodic changes throughout the menstrual cycle (**Figure 6C**). Among these, NK1 was the predominant population in the endometrium during the proliferative and secretory phases, while the percentage of NK2 cells with high expression of chemokines peaked during the secretory phase and then declined, and the main NK cells of the menstrual endometrium were *CD16*^high^*IFNG*^high^*GZMB*^high^*PRF1*^high^ NK4 cells, which are involved in immune defenses during menstruation (**Figure 6C**-**D**, and **Figure S11A**). In tumor microenvironment, a heightened capacity for glucose metabolism through glycolysis supports NK cells with the greatest cytotoxic capacity 33. However, the cytotoxicity of endometrial *LGALS1*^+^ *LDHA*^+^ NK3 cells with an activated glycolysis was not strong (**Figure 6D**, **Figure S11A**-**B**), which should be the progenitor cells of *CD49a*^+^*CD103*^-^*CD39*^+^*ITGB1*^-^ dNK1 cells with high levels of granule proteins, activated glycolytic metabolism and high expression of KIR genes 14. LGALS1, also known as galectin 1, is an important lectin with major functions in embryo implantation, modulation of maternal immune responses and placentation 34, suggesting that NK3 cells particularly interact with embryonic trophoblasts.

More importantly, *CD49a*^+^*CXCR4*^+^*CCR9*^+^ NK2 cells should be recruited by four subsets of *CCL25*^+^*CXCL12*^+^FIBs and two subsets of EC (Ciliated EC and EC1), and further contribute to the infiltration of other immune cells (*CCR1*^+^*CCR5*^+^Mo/Mφ, *CCR1*^+^*CCR5*^+^T cells, *XCR1*^+^ B cells and *XCR1*^+^ mast cells) into the endometrium by high levels of various chemokines (e.g., CCL3, XCL1, and XCL2) (**Figure 6E**-**F**). Additionally, Ciliated EC, EC1, EC2 and EC3 are involved in the recruitment of *CX3CR1*^+^*CXCR1*^+^ NK4 and mast cells (**Figure 6E**). These results indicate that *CD49a*^+^*CXCR4*^+^ NK2 cells should be a core node for the regulation of FIB/EC infiltration in immune cells. Further FCM analysis showed that there was an aberrant percentage of NK2 and NK4 cells in RIF patients (**Figure 6G**), which contributes to an imbalance in the endometrial immune microenvironment.

### Restricted Secretory Abilities of EC1 and FIB1 Contribute to the Decrease of NK2 in RIF Patients

**Figure 7:**
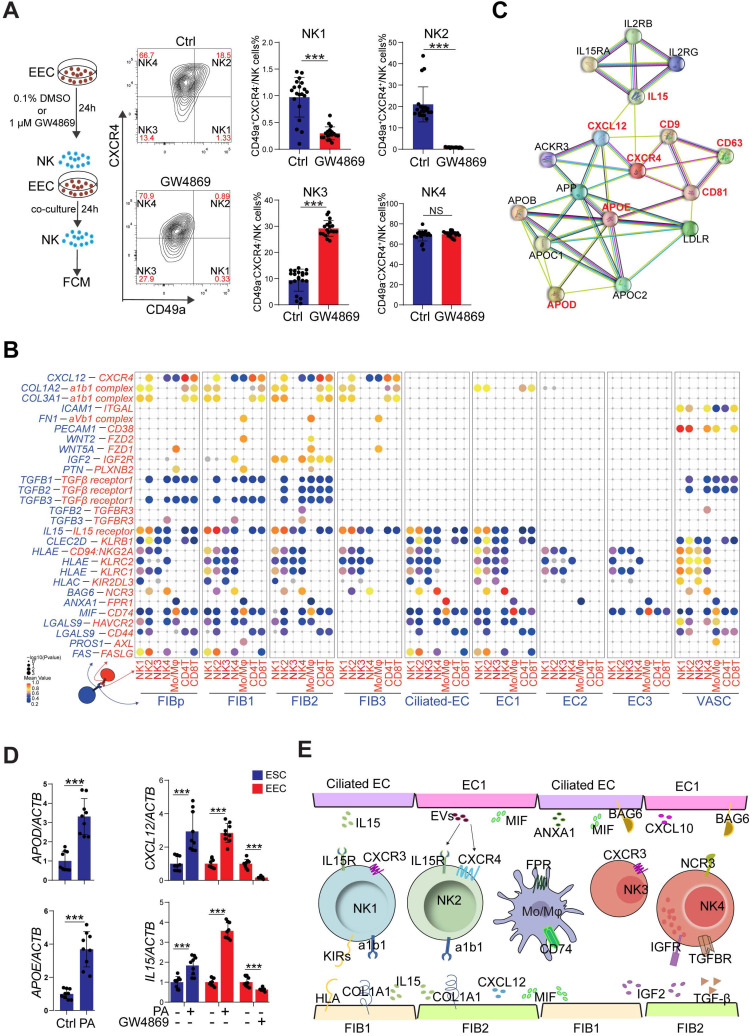
** Restricted secretory abilities of EC1 and FIB1 contribute to the decrease of NK2 cells of RIF patients. (A)** Flowchart overview of the experiment strategy of isolated endometrial NK cells and hEECs (left) in co-culture model (left). The hEECs were pre-treated with 0.1% DMSO (n = 20) or 1 µM GW4869 (n = 18) for 24 h, and then co-cultured with isolated endometrial NK cells for another 24 h, *in vitro*. The proportion of four sub-clusters of NK cells were analyzed by flow cytometry (right). Cells were initially gated within CD56^+^, and then CXCR4 and CD49a gating. Data were presented as mean ± SEM and analyzed by the t test (NS, no significance, ***, p < 0.001). **(B)** Overview of selected ligand-receptor interactions; P values indicated by circle size, scale on right. The means of the average expression level of interacting molecule 1 in cluster 1 and interacting molecule 2 in cluster 2 were indicated by color. **(C)** Protein-protein interaction network of exosome-related genes, APOD/APOE, CXCL12 and IL15. **(D)** Primary ESCs were treated with the vehicle or 10 μM PA for 48h, *in vitro*. The hEECs were treated with the vehicle or 10 µM PA for 48 h, or 0.1% DMSO or 1 µM GW4869 for 24 h, *in vitro*. The mRNA expression levels of these genes in ESCs and hEECs were then measured by qRT-PCR (n = 9). Data were presented as mean ± SEM and analyzed by t test (***, p < 0.001). **(E)** Schematic cell interactions in the endometrium. Cell interaction of endometrial non-immune cells (FIB and EC, ligands) and immune cells (NK, Mo/Mφ, receptors). The pathway and function of cell interactions were mainly involved in cell adhesion, cell migration and immunomodulation.

To systematically study the interactions of endometrial cells at the WOI, we developed a repository of ligand-receptor interacting pairs, representing a complex regulatory network by intercellular communication analysis (**Figure S12A**-**B**). Notably, the most intense crosstalk between EC1/Ciliated-EC and NK1/NK2 (**Figure S13A**-**B**). In addition to acting as messengers between the uterus and embryo, EVs have also been reported in recent years to be involved in immune regulation in tumor environments 35, 36. To further evaluate the potential role of EVs derived from epithelial cells on endometrial NK cell, GW4869, an inhibitor of exosome biogenesis/release, was used to treat hEECs for 24 h, and then co-cultured with primary isolated endometrial NK cells,* in vitro* (**Figure 7A**). The percentage of NK1 (nearly 1%) and NK2 (nearly 20%) after 48 h *in vitro* culture significantly decreased, as well as an obvious increase in NK4 (above 60%), suggesting that the local endometrial environment contributes to the survival, proliferation and/or differentiation of NK1 and NK2 (**Figure S13C**-**D**). Further observation showed that GW4869-pretreated hEEC led to a marked decrease in NK2 cells and an increase in NK3 cells in the co-culture system compared with control hEEC (**Figure 7A**), suggesting that exosomes released by EEC contributes to the survival and proliferation of resident NK1 and NK2 cells in the endometrium.

Interestingly, the CellPhoneDB results predicted that *CXCL12*/*CXCR4* and *IL15*/*IL15 receptor* are involved in the regulation of EC/FIB in the NK cells, especially the NK2 cells (**Figure 7B**), and these ligand-receptors are important factors for the recruitment, residence and proliferation of endometrial NK cells 37-39. It has been reported that progesterone regulates NK cells by IL-15 40, 41. EVs are enriched in certain types of lipids compared with their parent cells. For example, vesicles are enriched in cholesterol and saturated fatty acids 29, 42. Notably, both marker genes of exosomes and *APOD/APOE* were predicted to be associated with *CXCL12/CXCR4* and *IL15* (**Figure 7C**). We found that GW4869 down-regulated the expression of *CXCL12* and *IL15* in hEEC. Contrastingly, exposure to palmitic acid (PA) up-regulated the expression of *CXCL12* and *IL15* in hEEC and primary ESC *in vitro*, as well as *APOD/APOE* in ESC (**Figure 7D**). These findings indicate that EC1 with rich exosome and FIB1 with high levels of APOD/APOE are involved in the recruitment and survival of NK2 by secreting CXCL12 and IL15 (**Figure 7E**). The absence of EC1, and decreased levels of CXCL12 and IL15 in ESC contribute to the abnormal decrease of NK2 in the endometrium of RIF patients.

### Cell Communication Networks of Stromal Cells, Epithelial, and Immune Cells Contribute to Widespread Decidualization Features at the WOI

**Figure 8:**
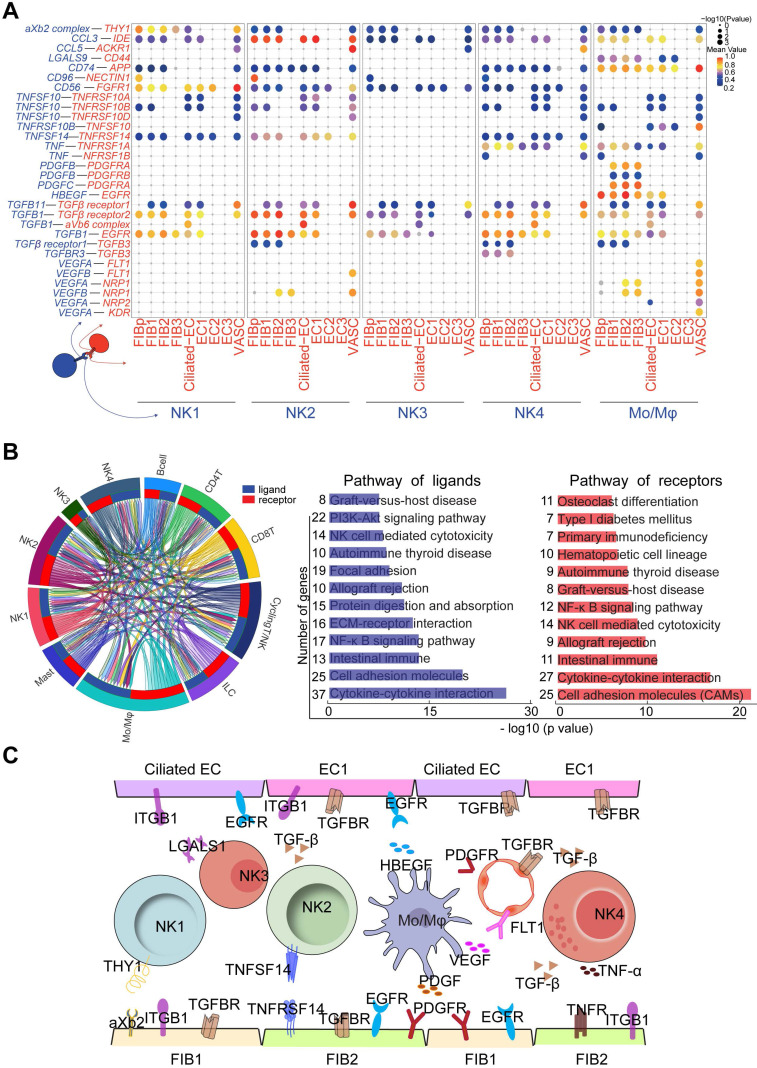
** Cell communication networks of FIB, EC and immune cells contribute to widespread decidualization features at the WOI. (A)** Overview of selected ligand-receptor interactions; P values indicated by circle size, and scale on right. The means of the average expression level of interacting molecule 1 in cluster 1 and interacting molecule 2 in cluster 2 were indicated by color. **(B)** Circos plot of interaction network for endometrial immune cells (left). The ribbons connect each ligand to the assigned receptors. The color of a ribbon is consistent with the color at the receptor side to ligand side. And ligand and receptor genes involved in each cluster were selected to perform pathway analysis (right). Pathway with P < 0.05 are shown. Gene number of each pathway is listed on the left. P value is shown as -log 10 (P value). **(C)** Cell interaction of immune cells (NK, Mo/Mφ, ligands), endometrial non-immune cells (FIB, EC and VASCs, receptors). Pathway and function of cell interactions were mainly involved in cell adhesion, cell growth and differentiation, tissue modeling, inflammatory response and angiogenesis.

In the endometrium, NK1 and NK2 have high levels of *NCAM1* (also known as CD56, which is highly expressed in decidual NK cells during early pregnancy), *CD49a* and *EOMES* (**Figure 8A**) and relatively low levels of *CD16*, *INFG*, and *GZMB*, suggesting that NK1 and NK2 are the progenitor cells of decidual NK cells 14, 43. Our previous work showed that TNFSF14 (also known as LIGHT) promotes cell adhesion and tissue remodeling of decidual stromal cells by interacting with TNF4SF14 (also known as HVEM) and further activating MMP9 signaling 32. Therefore, *TNFSF14*^+^ NK2 cells are beneficial to *TNF4SF14*^+^FIB/EC adhesion and NK2 cell residence in the endometrium at the WOI (**Figure 8A** and **8C**). In addition to promoting the infiltration and adhesion of NK1 and NK2 in the endometrium, FIBp, FIB1 and FIB2 were predicted to promote the differentiation of NK4 cells into decidual-like NK cells by *TGFB* (**Figure 7B** and** 7E**) 44. Furthermore, NK4 and Mo/Mφ-derived *TNF* were associated with moderate inflammation required for implantation (**Figure 8C**) 45.

Four subsets of FIBs were also involved in the adhesion and residence of Mo/Mφ via a variety of integrins and *LGALS9*/*CD44* (**Figure 8A**). Macrophage migration inhibitory factor (MIF) and IGF2 play critical roles in regulating inflammatory and anti-inflammatory properties of macrophage, respectively 46, 47. Here, we predicted that *MIF* and *IGF-2* derived from FIB2 and FIB1 regulated the homeostasis of endometrial Mo/Mφ (**Figure 7E** and** 8A**), which would prepare for moderate inflammation during embryo implantation and rapid transformation of maternal-fetal immune tolerance during early pregnancy. In turn, *TGFB*, *PDGFB* and *HBEGF* derived from endometrial Mo/Mφ and or NK cells (i.e., NK2 cell) accelerated the decidualization of FIB and differentiation of Ciliated EC, EC1 and EC2, and trophoblast invasion (**Figure 8A** and** 8C**) 48-51. Notably, endometrial Mo/Mφ and NK2 cells are considered to participate in angiogenesis and vascularization through the interaction of VEGF and its receptors (**Figure 8A** and** 8C**) 52-54. Additionally, close crosstalk between NK cells and other immune cells (e.g., Mo/Mφ) was observed, including the regulation of focal adhesion, ECM-receptor interaction, cytokine-cytokine interaction, NK cell-mediated cytotoxicity, and primary immunodeficiency (**Figure 8B**). Therefore, the proper proportion and function of the four major cellular subsets (FIB, EC, IC and VASC) contribute to the homeostasis and endometrial receptivity at the WOI through precise and orderly intercellular interactions. The subset imbalance of these cells (e.g, EC1, and NK2) was predicted to result in the pathogenesis of RIF with an aberrant cell communication network.

## Discussion

Implantation of the embryo into the uterine endometrium is one of the most finely-regulated processes that leads to a successful pregnancy. Disorders of endometrial receptivity and shifted window of receptivity are considered important etiologies for unexplained RIF; therefore, exploring the normal and pathological cell subsets and cell interaction mechanisms of the endometrium at the WOI is particularly critical for the diagnosis and treatment of explained RIF 4. Recent studies on the human endometrium have been limited to physiological states 5, 12, a single cell type 55, or a single patient with uterine leiomyoma 56. Herein, we focused on the human endometrium at the time of embryo transfer, and further identified cell subset changes and cell communication networks in the human endometrium, which were supported by multiple healthy and RIF biological replicates at the WOI and across the menstrual cycle.

Herein, we defined four major FIB populations (FIBp, FIB1, FIB2, and FIB3). Pseudotime analysis and further verification showed that FIBp with high proliferative activity, and FIB3 cells located in the end of single-cell trajectory mainly existed in the endometrium during proliferative and premenstrual phases, respectively. As the largest proportion of FIBs at the WOI, FIB1 and FIB2 displayed better endometrial receptivity for the opening of WOI, and promoted the recruitment, residence and proliferation of NK cells by the collagens/integrins, CXCL12/CXCR4, CCL25/CCR9 and IL15/IL15R interactions, contributing to the dominance of NK1 and NK2 cells in the endometrium at the WOI. FIB1 and/or FIB2-derived TGFB, and IGF2 signaling are also involved in the differentiation and cytotoxicity regulation of NK4 cells 57. Additionally, FIBs are involved in the recruitment and infiltration of other immune cells, including mast cells, ILC and T/NKp cells 57, 58 to build an immune microenvironment conducive to embryo implantation.

The Ciliated epithelium, the foreword place of embryo implantation, has been identified, but its characteristics and function remain largely undefined. Here, we observed that Ciliated-ELE exhibited cilium movement, organelle organization, and smoothened signaling pathways. Moreover, a novel group of EC, EC1, with powerful exosomes secretion was identified. The release of exosomes by EC1 should be dependent on progesterone/autophagy signaling and IGF-1 produced by FIB2 and FIB3. More importantly, Ciliated-EC, EC1, FIB1 and FIB2 work together to open the WOI, promote embryo adhesion and implantation by good endometrial receptivity, and trigger the rapid transformation of the immune microenvironment from moderate inflammation during embryo implantation to maternal-fetal immune tolerance during early pregnancy. These processes are dependent on the regulation of NK and Mo/Mφ activation by HLAs/KIRs and BAG6/NCR3 59 interactions, and MIF/CD74 and IGF2/IGF2R, respectively.

Notably, NK cells also exhibit dynamic and periodic changes throughout the menstrual cycle. As the potential progenitor cells of decidual NK cells, tissue resident NK1 and NK2 cells expressing *CD49a* and *EOMES* were dominant in the endometrium at the WOI, especially the NK2 cells. Decidual *CD49a^+^EOMES^+^* NK cells have been reported to promotes fetal growth during early pregnancy, and CD49a^+^EOMES^+^ NK cells in menstrual blood and decidua are associated with unexplained recurrent spontaneous abortion 60. The recruitment and survival of NK2 cells are dependent on exosomes secreted by EC1, and CXCL12/IL15 produced by FIB1. Interestingly, the NK2 cells are predicted to promote cellular adhesion and tissue remodeling in FIB/EC by TNFSF14/TNFRSF14 and MMP9 signaling. More importantly, the NK2 cells may act as an intermediate link between FIB/EC and other immune cell (e.g., Mo/Mφ) infiltration, which deserves further study. NK2 cells, together with Mo/Mφ, are predicted to accelerate the decidualization and differentiation of FIB/EC to open the WOI, angiogenesis and vascularization, and trophoblast invasion by the production of TGFB, PDGFB, HBEGF and VEGF. However, the specific mechanism needs to be studied further.

Additionally, this study has several limitations. On the one hand, it is challenging to digest endometrial glands into single cells. The number of epithelial cells obtained in this study was relatively limited and needs to be improved. However, the sample size of this study is limited, and the etiology of RIF was complex. Additionally, there are marked differences in individuals of RIF patients, which need to be further verified by large sample studies.

Notably, several classic marker genes (e.g, PRL and LIF) for endometrial receptivity and decidualization were detected at low levels, which was also reported by Wang *et al.*, 5. This suggests that embryo implantation plays an important role in rapidly accelerating decidualization. Benign and effective interactions between the mother and fetus during endometrial differentiation for embryo implantation should be emphasized. More interestingly, there was significantly decreased expression of autophagy, exosomes and senescence-related genes in both FIB and EC from RIF patients, suggesting that appropriate autophagy, exosomes and senescence are important characteristics of the endometrium for embryo implantation during the WOI time 61-65. Therefore, abnormally low levels of these functional genes should contribute to the pathogenesis of RIF, and further research needs to focus on their value in the prediction and therapy of RIF.

## Conclusion

Collectively, the proper proportion, precise and orderly intercellular communication of constitutive cells (FIB, EC, IC and VASC) are beneficial for endometrial receptivity and the opening of WOI. The abnormal expression of endometrial receptivity and immune regulation-related molecules in FIBs, and the percentages of EC1 and NK2 cells are associated with RIF. The potential mechanisms for these disorders include progesterone insensitivity-induced autophagy and secretion limitation of EC1, deficiency of the APOD/APOE/CXCL12/IL15 axis-mediated poor decidualization, NK2 infiltration and survival, and incorrect selection for embryo transfer time of IVF. Therefore, a comprehensive and detailed analysis of the subpopulations of cells in the endometrium will help us identify possible causes of RIF and pave the way toward new diagnostic and therapeutic strategies for RIF, for example, choosing a more suitable time for embryo transfer to avoid a shifted window of receptivity or poor endometrial receptivity.

## Supplementary Material

Supplementary figures and tables.

## Figures and Tables

**Table 1:** Baseline Characteristics

	Ctrl (n = 3)	RIF (n = 6)	P-value^a^
Age (years) (median ± IQR)	35 (33.5 - 35.5)	33 (31.5 - 34)	0.267
BMI (median ± IQR)	20.03 (19.87 - 21.46)	21.37 (20.84 - 21.59)	0.530
Endometrial thickness (mm) (median ± IQR)	8 (6.65 - 9)	9.15 (8.5 - 10.025)	0.21
LH + day	7	7	-
Live births [mean (range)]	1 (0 - 1)	0 (0 - 0)	0.0325
Times of IVF-ET (median ± IQR)	1 (0.5 - 1)	4 (3 - 5.75)	0.0176
Embryos quality grade	II	II	-
Menstrual cycling	Regularly (6-7 days every 28-30 days)	Regularly (6-7 days every 28-30 days)	-
Endocrine metabolic abnormalities	Without	Without	-

a: P-value was calculated by two-tailed Mann Whitney test for non-normally distributed data.

**Table 2:** Inclusion and exclusion criteria

	Ctrl	RIF
**Inclusion criteria**		
Age (years)	25 - 36	25 - 36
Fertility history^#^	Yes	No
Infertility reasons	mechanical obstruction of fallopian tube, infertility due to male factors	unexplained
Times of IVF-ET	≤ 1	≥ 3
**Exclusion criteria**		
1. Recent contraception (intrauterine device usage in past 3 months; hormonal contraceptives in past 3 months); 2. Endocrine metabolic abnormalities (i.e., polycystic ovary syndrome, diabetes, insulin resistance, hypothyroidism); 3. Genetic abnormalities, severe adenomyosis or endometriosis, severe hydrosalpinx, moderate to severe intrauterine adhesions, uterine malformations, recurrent miscarriage, thrombosis and autoimmune diseases, BMI >30; 4. Unwilling to sign informed consent.		

#: Fertility history was defined as previous pregnancies, including the number of full-term births, preterm births and miscarriages, and the number of surviving children.

## Abbreviations

Ctrl: control
EC: epithelial cells
ECM: extracellular matrix
EEC: endometrial epithelial cell
ESC: endometrial stomal cells
Evs: extracellular vesicles
FDR: false discovery rate
FIB: fibroblast-like cells
GO: gene ontology
IC: immune cells
IGFBP: Insulin Like Growth Factor Binding Protein
IQR: Inter-quartile range
IVF-ET: *in vitro* fertilization-embryo transfer
MFC: myofibroblasts
Mo/Mφ: monocyte/macrophage
MPA: medroxyprogesterone acetate
MUC1: mucin 1
PA: palmitic acid
qRT-PCR: Quantitative Real-Time Polymerase Chain Reaction
RIF: recurrent implantation failure
scRNA-seq: single-cell RNA sequencing
VASC: vascular cells
WOI: window of implantation

## References

[1] Diedrich K, Fauser BC, Devroey P, Griesinger G, Evian Annual Reproduction Workshop G (2007). The role of the endometrium and embryo in human implantation. Hum Reprod Update.

[2] Coughlan C, Ledger W, Wang Q, Liu F, Demirol A, Gurgan T (2014). Recurrent implantation failure: definition and management. Reprod Biomed Online.

[3] Wu F, Chen X, Liu Y, Liang B, Xu H, Li TC (2018). Decreased MUC1 in endometrium is an independent receptivity marker in recurrent implantation failure during implantation window. Reprod Biol Endocrinol.

[4] Moustafa S, Young SL (2020). Diagnostic and therapeutic options in recurrent implantation failure. F1000Res.

[5] Wang W, Vilella F, Alama P, Moreno I, Mignardi M, Isakova A (2020). Single-cell transcriptomic atlas of the human endometrium during the menstrual cycle. Nat Med.

[6] Lessey BA, Young SL (2019). What exactly is endometrial receptivity?. Fertil Steril.

[7] Achache H, Revel A (2006). Endometrial receptivity markers, the journey to successful embryo implantation. Hum Reprod Update.

[8] Ruiz-Alonso M, Blesa D, Diaz-Gimeno P, Gomez E, Fernandez-Sanchez M, Carranza F (2013). The endometrial receptivity array for diagnosis and personalized embryo transfer as a treatment for patients with repeated implantation failure. Fertil Steril.

[9] Diaz-Gimeno P, Ruiz-Alonso M, Blesa D, Bosch N, Martinez-Conejero JA, Alama P (2013). The accuracy and reproducibility of the endometrial receptivity array is superior to histology as a diagnostic method for endometrial receptivity. Fertil Steril.

[10] Saxtorph MH, Hallager T, Persson G, Petersen KB, Eriksen JO, Larsen LG (2020). Assessing endometrial receptivity after recurrent implantation failure: a prospective controlled cohort study. Reprod Biomed Online.

[11] Ben Rafael Z (2021). Endometrial Receptivity Analysis (ERA) test: an unproven technology. Hum Reprod Open.

[12] Garcia-Alonso L, Handfield L-F, Roberts K, Nikolakopoulou K, Fernando RC, Gardner L (2021). Mapping the temporal and spatial dynamics of the human endometrium *in vivo* and *in vitro*. Nature Genetics.

[13] Chen S, Zhou Y, Chen Y, Gu J (2018). fastp: an ultra-fast all-in-one FASTQ preprocessor. Bioinformatics.

[14] Vento-Tormo R, Efremova M, Botting RA, Turco MY, Vento-Tormo M, Meyer KB (2018). Single-cell reconstruction of the early maternal-fetal interface in humans. Nature.

[15] Yaari G, Bolen CR, Thakar J, Kleinstein SH (2013). Quantitative set analysis for gene expression: a method to quantify gene set differential expression including gene-gene correlations. Nucleic Acids Res.

[16] Li MQ, Li HP, Meng YH, Wang XQ, Zhu XY, Mei J (2012). Chemokine CCL2 enhances survival and invasiveness of endometrial stromal cells in an autocrine manner by activating Akt and MAPK/Erk1/2 signal pathway. Fertil Steril.

[17] Zhou WJ, Yang HL, Mei J, Chang KK, Lu H, Lai ZZ (2022). Fructose-1,6-bisphosphate prevents pregnancy loss by inducing decidual COX-2(+) macrophage differentiation. Sci Adv.

[18] Hannan NJ, Evans J, Salamonsen LA (2011). Alternate roles for immune regulators: establishing endometrial receptivity for implantation. Expert Rev Clin Immunol.

[19] Cha J, Sun X, Dey SK (2012). Mechanisms of implantation: strategies for successful pregnancy. Nat Med.

[20] Sharkey AM, Xiong S, Kennedy PR, Gardner L, Farrell LE, Chazara O (2015). Tissue-Specific Education of Decidual NK Cells. J Immunol.

[21] Feyaerts D, Kuret T, van Cranenbroek B, van der Zeeuw-Hingrez S, van der Heijden OWH, van der Meer A (2018). Endometrial natural killer (NK) cells reveal a tissue-specific receptor repertoire. Hum Reprod.

[22] Berneau SC, Ruane PT, Brison DR, Kimber SJ, Westwood M, Aplin JD (2019). Investigating the role of CD44 and hyaluronate in embryo-epithelial interaction using an *in vitro* model. Mol Hum Reprod.

[23] Latifi Z, Fattahi A, Ranjbaran A, Nejabati HR, Imakawa K (2018). Potential roles of metalloproteinases of endometrium-derived exosomes in embryo-maternal crosstalk during implantation. J Cell Physiol.

[24] Monsivais D, Nagashima T, Prunskaite-Hyyrylainen R, Nozawa K, Shimada K, Tang S (2021). Endometrial receptivity and implantation require uterine BMP signaling through an ACVR2A-SMAD1/SMAD5 axis. Nat Commun.

[25] Murphy CR (2004). Uterine receptivity and the plasma membrane transformation. Cell Res.

[26] Garrido-Gomez T, Ruiz-Alonso M, Blesa D, Diaz-Gimeno P, Vilella F, Simon C (2013). Profiling the gene signature of endometrial receptivity: clinical results. Fertil Steril.

[27] Park Y, Jung JG, Yu ZC, Asaka R, Shen W, Wang Y (2021). A novel human endometrial epithelial cell line for modeling gynecological diseases and for drug screening. Lab Invest.

[28] Hild-Petito S, Fazleabas AT, Julian J, Carson DD (1996). Mucin (Muc-1) expression is differentially regulated in uterine luminal and glandular epithelia of the baboon (Papio anubis). Biol Reprod.

[29] Simon C, Greening DW, Bolumar D, Balaguer N, Salamonsen LA, Vilella F (2018). Extracellular Vesicles in Human Reproduction in Health and Disease. Endocr Rev.

[30] Machtinger R, Laurent LC, Baccarelli AA (2016). Extracellular vesicles: roles in gamete maturation, fertilization and embryo implantation. Hum Reprod Update.

[31] Blois SM, Klapp BF, Barrientos G (2011). Decidualization and angiogenesis in early pregnancy: unravelling the functions of DC and NK cells. J Reprod Immunol.

[32] Lu H, Yang HL, Zhou WJ, Lai ZZ, Qiu XM, Fu Q (2020). Rapamycin prevents spontaneous abortion by triggering decidual stromal cell autophagy-mediated NK cell residence. Autophagy.

[33] Cong J, Wang X, Zheng X, Wang D, Fu B, Sun R (2018). Dysfunction of Natural Killer Cells by FBP1-Induced Inhibition of Glycolysis during Lung Cancer Progression. Cell Metab.

[34] Barrientos G, Freitag N, Tirado-Gonzalez I, Unverdorben L, Jeschke U, Thijssen VL (2014). Involvement of galectin-1 in reproduction: past, present and future. Hum Reprod Update.

[35] Zhou WJ, Zhang J, Xie F, Wu JN, Ye JF, Wang J (2021). CD45RO(-)CD8(+) T cell-derived exosomes restrict estrogen-driven endometrial cancer development via the ERbeta/miR-765/PLP2/Notch axis. Theranostics.

[36] Pepe G, Braga D, Renzi TA, Villa A, Bolego C, D'Avila F (2017). Self-renewal and phenotypic conversion are the main physiological responses of macrophages to the endogenous estrogen surge. Sci Rep.

[37] Hanna J, Wald O, Goldman-Wohl D, Prus D, Markel G, Gazit R (2003). CXCL12 expression by invasive trophoblasts induces the specific migration of CD16- human natural killer cells. Blood.

[38] Lu H, Jin LP, Huang HL, Ha SY, Yang HL, Chang RQ (2020). Trophoblast-derived CXCL12 promotes CD56(bright) CD82(-) CD29(+) NK cell enrichment in the decidua. Am J Reprod Immunol.

[39] Kitaya K, Yamaguchi T, Honjo H (2005). Central role of interleukin-15 in postovulatory recruitment of peripheral blood CD16(-) natural killer cells into human endometrium. J Clin Endocrinol Metab.

[40] Verma S, Hiby SE, Loke YW, King A (2000). Human decidual natural killer cells express the receptor for and respond to the cytokine interleukin 15. Biol Reprod.

[41] Kitaya K, Yasuda J, Yagi I, Tada Y, Fushiki S, Honjo H (2000). IL-15 expression at human endometrium and decidua. Biol Reprod.

[42] Trajkovic K, Hsu C, Chiantia S, Rajendran L, Wenzel D, Wieland F (2008). Ceramide triggers budding of exosome vesicles into multivesicular endosomes. Science.

[43] Fu B, Zhou Y, Ni X, Tong X, Xu X, Dong Z (2017). Natural Killer Cells Promote Fetal Development through the Secretion of Growth-Promoting Factors. Immunity.

[44] Keskin DB, Allan DS, Rybalov B, Andzelm MM, Stern JN, Kopcow HD (2007). TGFbeta promotes conversion of CD16+ peripheral blood NK cells into CD16- NK cells with similarities to decidual NK cells. Proc Natl Acad Sci U S A.

[45] Salama KM, Alloush MK, Al Hussini RM (2020). Are the cytokines TNF alpha and IL 1Beta early predictors of embryo implantation? Cross sectional study. J Reprod Immunol.

[46] Du L, Lin L, Li Q, Liu K, Huang Y, Wang X (2019). IGF-2 Preprograms Maturing Macrophages to Acquire Oxidative Phosphorylation-Dependent Anti-inflammatory Properties. Cell Metab.

[47] Calandra T, Roger T (2003). Macrophage migration inhibitory factor: a regulator of innate immunity. Nat Rev Immunol.

[48] Ni N, Li Q (2017). TGFbeta superfamily signaling and uterine decidualization. Reprod Biol Endocrinol.

[49] Li Y, Yan J, Chang HM, Chen ZJ, Leung PCK (2021). Roles of TGF-beta Superfamily Proteins in Extravillous Trophoblast Invasion. Trends Endocrinol Metab.

[50] Annunziata M, Luque RM, Duran-Prado M, Baragli A, Grande C, Volante M (2012). Somatostatin and somatostatin analogues reduce PDGF-induced endometrial cell proliferation and motility. Hum Reprod.

[51] Xie H, Wang H, Tranguch S, Iwamoto R, Mekada E, Demayo FJ (2007). Maternal heparin-binding-EGF deficiency limits pregnancy success in mice. Proc Natl Acad Sci U S A.

[52] Gamliel M, Goldman-Wohl D, Isaacson B, Gur C, Stein N, Yamin R (2018). Trained Memory of Human Uterine NK Cells Enhances Their Function in Subsequent Pregnancies. Immunity.

[53] Sugino N, Kashida S, Karube-Harada A, Takiguchi S, Kato H (2002). Expression of vascular endothelial growth factor (VEGF) and its receptors in human endometrium throughout the menstrual cycle and in early pregnancy. Reproduction.

[54] Kim M, Park HJ, Seol JW, Jang JY, Cho YS, Kim KR (2013). VEGF-A regulated by progesterone governs uterine angiogenesis and vascular remodelling during pregnancy. EMBO Mol Med.

[55] Krjutskov K, Katayama S, Saare M, Vera-Rodriguez M, Lubenets D, Samuel K (2016). Single-cell transcriptome analysis of endometrial tissue. Hum Reprod.

[56] Tan HX, Yang SL, Li MQ, Wang HY (2020). Autophagy suppression of trophoblast cells induces pregnancy loss by activating decidual NK cytotoxicity and inhibiting trophoblast invasion. Cell Commun Signal.

[57] Menzies FM, Shepherd MC, Nibbs RJ, Nelson SM (2011). The role of mast cells and their mediators in reproduction, pregnancy and labour. Hum Reprod Update.

[58] Li M, Gao Y, Yong L, Huang D, Shen J, Liu M (2017). Molecular signature and functional analysis of uterine ILCs in mouse pregnancy. J Reprod Immunol.

[59] Pogge von Strandmann E, Simhadri VR, von Tresckow B, Sasse S, Reiners KS, Hansen HP (2007). Human leukocyte antigen-B-associated transcript 3 is released from tumor cells and engages the NKp30 receptor on natural killer cells. Immunity.

[60] Tong X, Gao M, Du X, Lu F, Wu L, Wei H (2021). Analysis of uterine CD49a(+) NK cell subsets in menstrual blood reflects endometrial status and association with recurrent spontaneous abortion. Cell Mol Immunol.

[61] Oestreich AK, Chadchan SB, Popli P, Medvedeva A, Rowen MN, Stephens CS (2020). The Autophagy Gene Atg16L1 is Necessary for Endometrial Decidualization. Endocrinology.

[62] Matsubara M, Unagami H, Totsune K, Sato H, Kikuta Y, Ogawa M (1988). Long-term CaCO3 treatment of chronic hemodialysis patients: an attempt to prevent aluminum osteopathy. ASAIO Trans.

[63] Oestreich AK, Chadchan SB, Medvedeva A, Lydon JP, Jungheim ES, Moley KH (2020). The autophagy protein, FIP200 (RB1CC1) mediates progesterone responses governing uterine receptivity and decidualizationdagger. Biol Reprod.

[64] Rawlings TM, Makwana K, Taylor DM, Mole MA, Fishwick KJ, Tryfonos M (2021). Modelling the impact of decidual senescence on embryo implantation in human endometrial assembloids. Elife.

[65] Segura-Benitez M, Carbajo-Garcia MC, Corachan A, Faus A, Pellicer A, Ferrero H (2022). Proteomic analysis of extracellular vesicles secreted by primary human epithelial endometrial cells reveals key proteins related to embryo implantation. Reprod Biol Endocrinol.

