# Supplementary material for: Single-cell transcriptome profiling of the human endometrium of patients with recurrent implantation failure
Source: Theranostics. 2022 Sep 6;12(15):6527–47. doi: 10.7150/thno.74053 (PMC9516226; doi:10.7150/thno.74053)
Supplement: Supplementary file 1 — Supplementary figures and tables. [file thnov12p6527s1.pdf]

Supplementary information

Exosomes enrichment, characterization, purification and analysis

After passage 3, hEECs were cultured to 80-90% confluence in complete medium (MEM containing 10% fetal bovine serum and 1% penicillin/streptomycin). Then, the complete medium was replaced with serum-free medium. Fifteen bottles of T75 cell culture flasks were used for each isolation, meaning exosomes from 10×10^6 hEECs were isolated each time. Twenty-four hours later, the medium was collected, larger vesicles and debris were removed by centrifugations at 3,000 × g for 20 min, then filtration through 0.22 μm pore filters (TPP Techno Plastic Products AG, Trasadingen, Switzerland). For ultracentrifugation (UC), the conditioned medium (CM) was centrifuged for 1h at 110,000 g (Optima XPN-80 Ultracentrifuge, BECKMAN COULTER, Brea, California, United States) to pellet exosomes and washed with PBS by a centrifugation at 110,000 g subsequently. For the polyethylene glycol (PEG) precipitation method, PEG precipitation was performed at a final concentration of 10% PEG 6,000 (50% wt/vol; Merck Group, Darmstadt, Germany) and 75 mM NaCl. After incubation for at least 12 h at 4 °C, Exosomes were concentrated by centrifugation for 30min at 4,500 g. Exosome pellets were then resolved in PBS to a total volume of 30 mL and precipitated by UC for 1 h at 110,000 g and washed with PBS by centrifugation at 110,000 g subsequently. Then, exosomes were diluted in 500 μL of PBS, each microlitre contained 2×10^4 cell equivalent exosomes which yielded about 0.1 μg/μL protein and an average of 405 ± 186 particles/cell. Exosome pellets were resolved and stored at -80 °C until usage. For medium control the same amount of complete medium has been processed in the very same manner as for conditioned medium using the PEG method.

Transmission electron microscopy (TEM) was used to investigate the microstructure of EECs-exosomes. Briefly, formvar-coated TEM grids (copper, 150 hexagonal mesh, Science Services, Munich, Germany) were put on the top of a droplet of the respective exosome fraction and incubated for 10 min. Then, the grids were washed and incubated with ultrapure water. For contrast, the grids were incubated for 5min on droplets of uranylacetate-oxalate, followed by a 5-min incubation on droplets of a 1:9 dilution of 4% uranylacetate in 2% methylcellulose. After draining the methylcellulose from the grids using a filter paper and drying of the methylcellulose film as previously described, samples were imaged with a LEO912 transmission electron microscope (Carl Zeiss Microscopy, Oberkochen, Germany) and images were taken using an onaxis 2k CCD camera (TRS-STAR, Stutensee, Germany).

Nanoparticle tracking analyses were performed with the Nanosight platform (NanoSight LM10, Malvern Panalytical, Kassel, Germany) for exosomes characterization. As shown previously, 1:1,000 water-diluted samples were measured in duplicate. For iodixanol gradient separation, a discontinuous iodixanol gradient was prepared by diluting a stock solution of OptiPrepTM (60% w/v; STEMCELL Technologies, Vancouver, Canada) with 0.25 M sucrose/10 mM Tris, pH 7.5 to generate 50%, 20% and 10% w/v iodixanol solutions. exosomes obtained from equal amount of ADMSCs by UC or PEG were washed and resuspended in 3 mL of the aforementioned solution. With care, the discontinuous iodixanol gradient was generated by sequentially layering 3 mL each of 50%, 20% and 10% (w/v) iodixanol solutions. Three mL of resuspended CM was overlaid on the discontinuous iodixanol gradient and centrifuged for 12 h at 110,000 g. Fractions of 1.2 mL were collected from the top of the gradient. Each fraction (1 to 10) was diluted to 20 mL in PBS and recentrifuged at 110,000 g

for 2 h. The resulting pellets were resuspended in PBS and analyzed for particle concentration or exosomes markers. For UC and PEG combined with size-exclusion chromatography (SEC), the resuspended pellets from UC and PEG were loaded onto Exo-spin™ buffer columns (Cell Guidance Systems, Cambridge, UK) according to the manufacturer's instructions. The exosomes-containing fractions were pooled and concentrated using Amicon spin filters (Merck Group, Darmstadt, Germany).

To review GEO accession GSE183837:

Go to https://www.ncbi.nlm.nih.gov/geo/query/acc.cgi?acc=GSE183837

Enter token ypgnaguglbobpin into the box

Table S1 Antibodies for Flow Cytometry

Antibody	Manufactory	Cat No.
Brilliant violent (BV) 421 Anti-human CD45	BioLegend	368522
APC/Cyanine 7 (APC/Cy7) Anti-human HLA-DR	BioLegend	307618
Fluorescein isothiocyanate (FITC) Anti-human CD3	BioLegend	317306
BV605 Anti-human CD56	BioLegend	362538
Phycoerythrin (PE) Anti-human CD4	BioLegend	357404
APC Anti-human CD8	BioLegend	344722
APC Anti-human CXCR4	BioLegend	306510
PE/Cy7 Anti-human ITGA1	BioLegend	328312
PE/Cy7 Anti-human CD14	BioLegend	367112
PE/Cy7 Anti-human CD15	BioLegend	301924
PE Anti-human CD11B	BioLegend	393112
FITC Anti-human CD1C	BioLegend	331518
APC Anti-human Ki67	BioLegend	350514
PE/Cy7 Anti-human CD94	BioLegend	305516
BV605 Anti-human CD19	BioLegend	302244
APC Anti-human CD117	BioLegend	375204

Table S2 Gene primers for qRT-PCR

Name	Sequence (5' -> 3')
ACTB	Forwards: GCCGACAGGATGCAGAAGGAGATCA
	Reverse: AAGCATTTGCGGTGGACGATGGA
APOD	Forwards: CTTGGGAAGTGCCCCAATCC
	Reverse: TGCCGATGGCATAAACCAGG
APOE	Forwards: CTCAGGGGCCTCTAGAAAGA
	Reverse: CAGCGCAGGTAATCCCAAAAG
CXCL12	Forwards: TCAACACTCCAAACTGTGCCCTTC
	Reverse: TCCACTTTAGCTTCGGGTCAATGC
IL15	Forwards: CCATCCAGTGCTACTTGTGTTT
	Reverse: TCACCCAGTTGGCTTCTGTTTTAGG
ATG5	Forwards: GCTTCGAGTGTGTGGTTTGG
	Reverse: ACTTTGTCAGTTACCAACGTCA-
ATG7	Forwards: CAGTTTGCCCCTTTTAGTAGTGC
	Reverse: CCAGCCGATACTCGTTCAGC
BECN1	Forwards: ATCTAAGGAGCTGCCGTTATAC
	Reverse: CTCCTCAGAGTTAAACTGGGTT
MAP1LC3B	Forwards: TTATTCGAGAGCAGCATCCAACC
	Reverse: CCGTTCACCAACAGGAAGAAGG
mTOR	Forwards: GAGATACGCTGTCATCCCTTTA
	Reverse: CTGTATTATTGACGGCATGCTC
MUC1	Forwards: TGCCGCCGAAAGAACTACG
	Reverse: TGGGGTACTCGCTCATAGGAT
CK7	Forwards: TCCGCGAGGTCACCATTAAC
	Reverse: GCTCTGTCAACTCCGTCTCAT
EpCAM	Forwards: AATCGTCAATGCCAGTGTACTT
	Reverse: TCTCATCGCAGTCAGGATCATAA
ESR1	Forwards: CCCACTCAACAGCGTGTCTC
	Reverse: CGTCGATTATCTGAATTTGGCCT
PAX8	Forwards: ATCCGGCCTGGAGTGATAGG
	Reverse: TGGCGTTTGTAGTCCCCAATC

Table S3 Gene list of of six functions

Age	Sex hormone signaling	Immune pathway	Cell proliferation	Embryo implantation	ECM remodeling
APOD	F3	CD24	S100A13	CRABP2	COL6A1
TSPO	WFDC1	LGALS1	IGF1	ALDH1A2	ELN
IL15	ALDH1A2	HLA-C	TSC22D1	CST3	GSN
GPX1	KRT19	HLA-A	PTN	FBLN1	COL6A2
DCN	CD24	CXCL13	ALDH1A2	RPL29	COL3A1
SERPING1	TSPO	CXCL12	SFRP1	APOD	COL6A3
SOD2	C3	IL15	CLEC11A	SAT1	COL4A1
FOS	FOS	IL32	F3	HAND2	COL7A1
JUND	CST3	C1R	ECM1	RHOB	FN1
JUN	GPX1	C3	CXCL13	ID1	CD44
GSN	ANXA1	CEBPB	SAT1	JUN	COL18A1
COL3A1	STAT3	B2M	KLF4	KLF4	COL6A1
STAT3	SLIT3	IFI16	PLA2G2A	INSR	APP
SOCS3	JUND	GAL	ANXA1	EGR1	CYR61
SLC18A2	JUN	NFKBIZ	NFKBIA	APOLD1	TNC
	PDGFRB	ANXA1	JUND	COL4A1	LAMA2
	SOCS3	FOS	JUN	THSD7A	P3H2
		CFD	IGFBP3	FN1	COL21A1
		GBP1	ADAMTS1	COL18A1	CXCL12
		SERPING1	RARRES1		IL32
		IRS2	GAL		ADAM12
		ACTB	HSPA1B		RHOB
		HSP90AA1	SOD2		SPOCK1
		NFKBIA	HSPA1A		SPON1
		NR4A1	CST3		PCDH18
		JUN	NAMPT		LAMA3
		MCL1	CXCL12		MMP11
		JUND	IRS2		LAMC1
		TGM2	IL15		RND3
		PLA2G2A	MYC		
		EGR1	INSR		
		SEC61G	ID2		
		SEC61B	ATF3		
		PSMA7	ELN		
			CLK1		
			CYR61		
			STAT3		
			HGF		
			ZFP36L1		
			CENPF		

MKI67
PDGFRB
MIS18BP1
RAD21
CNTRL
ANLN
CENPE
CKAP5
ASPM
KNL1

Table S4. Information for Figure 3E

N.O.	Age (Years)	BMI	Menstrual cycling	Day of endometrial biopsy
53	30	22.95	6 days every 28 days	3th
54	32	21.48	5 days every 30 days	2th
56	30	20.69	7 days every 28 days	3th
57	30	22.43	10 days every 35 days	2th
59	30	20.08	6 days every 28 days	2th
60	31	22.83	6 days every 28 days	3th
67	29	21.48	6 days every 28 days	10th
68	30	22.43	6 days every 30 days	11th
69	29	20.55	4 days every 28 days	10th
70	37	21.48	7 days every 35 days	10th
71	33	20.57	6 days every 28 days	10th
72	34	26.03	6 days every 28 days	11th
116	33	20.17	6 days every 28 days	22th
117	31	27.64	5 days every 28 days	22th
118	26	20.96	7 days every 28 days	23th
120	32	21.51	6 days every 30 days	23th
121	32	18.42	6 days every 28 days	21th
123	27	19.72	6 days every 28 days	22th

Table S5. Information for Figure 5E

Group	N.O.	Age (Years)	BMI	Menstrual cycling	Day of endometrial biopsy
Ctrl	73	26	20.94	6 days every 28 days	22th
	74	22	20.96	7 days every 28 days	22th
	75	34	20.57	7 days every 28 days	22th
	74	31	23.58	6 days every 30 days	22th
	77	33	26.22	6 days every 28 days	22th
	60	31	27.64	6 days every 28 days	22th
RIF	1	29	21.34	6 days every 28 days	22th
	2	44	19.23	4 days every 28 days	22th
	3	31	21.48	7 days every 28 days	22th
	4	32	19.33	5 days every 30 days	22th
	5	33	21.51	6 days every 28 days	22th
	6	32	23.92	6 days every 28 days	22th

Figure legends

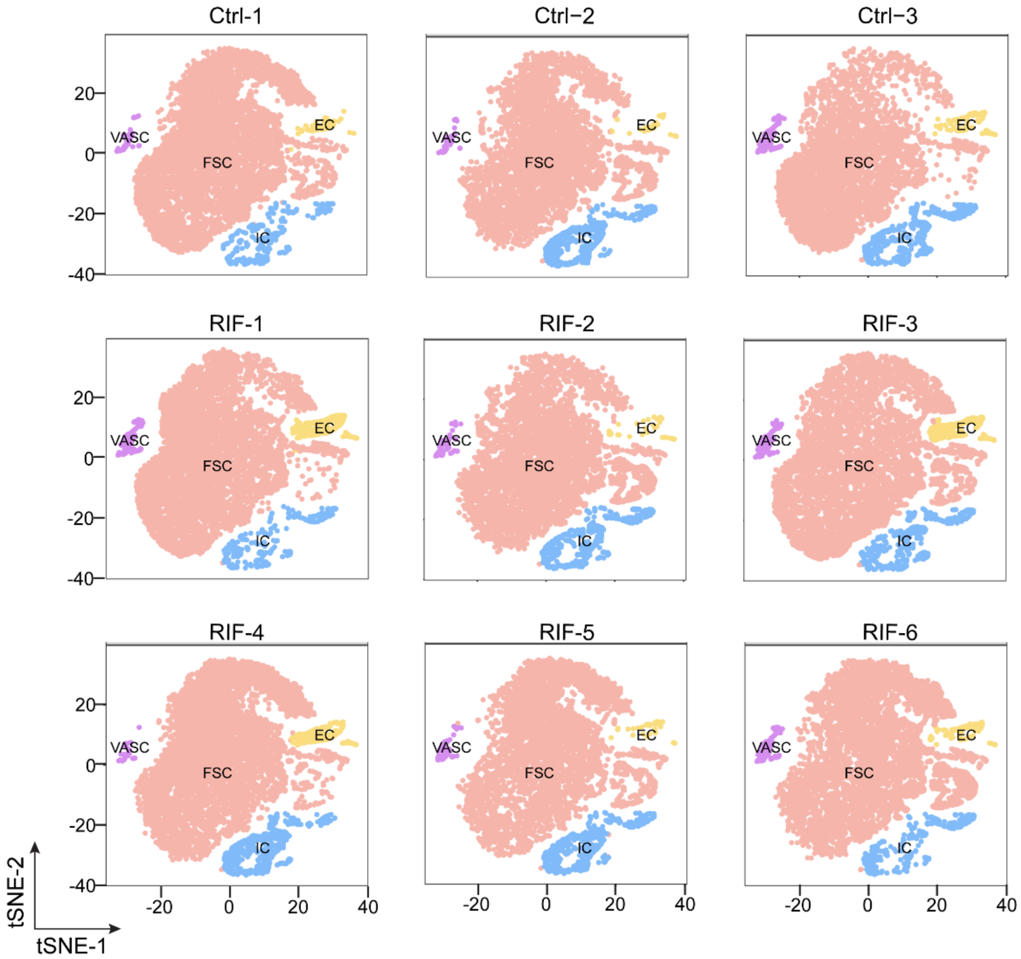

Figure S1 *t*-SNE map showing the distribution of 4 subsets of cells in different endometrium samples (3 healthy control and 6 RIF patients).

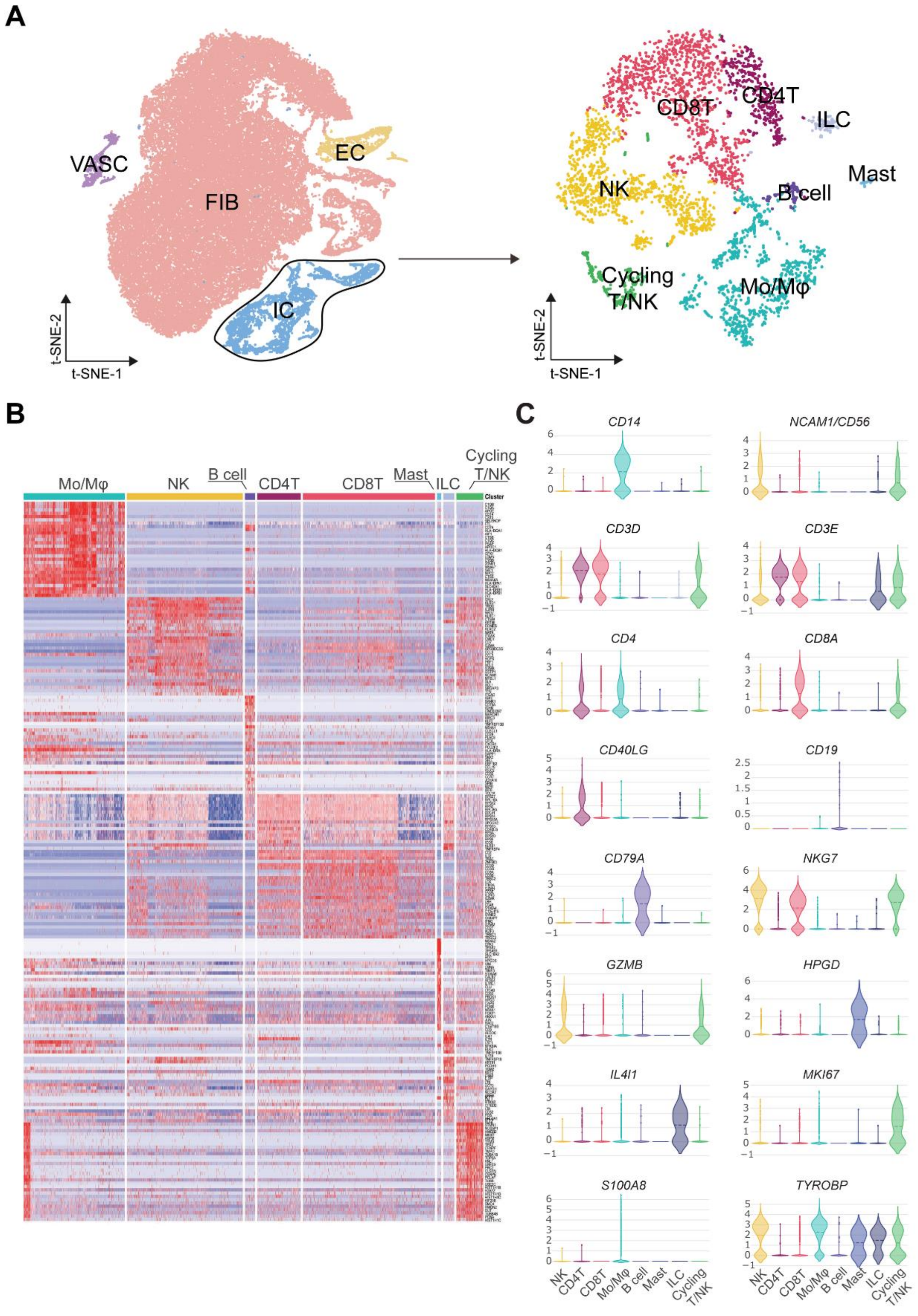

Figure S2 Subtypes of endometrial immune cell subtypes.

(**A**) t-SNE plots of IC cells (4790 cells), indicating eight main clusters.

(**B**, **C**) Heat map and violin plots showed the expression of differentially marker genes for each

cluster.

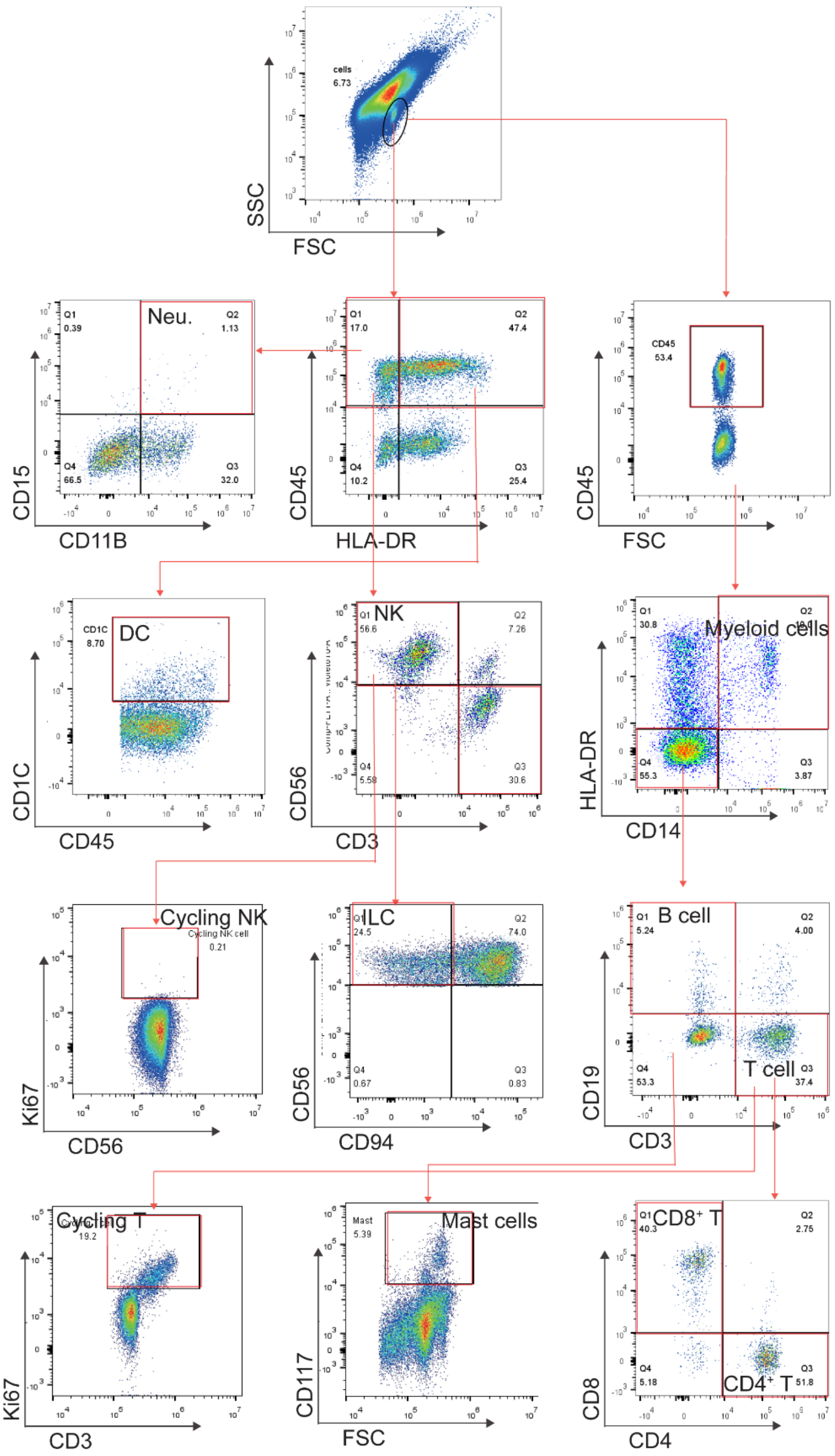

Figure S3 Characteristics of endometrial immune cell subsets by FCM

Gating strategy for a panel of 16 antibodies to analyze the different subsets of immune cells in endometrium from controls by flow cytometry.

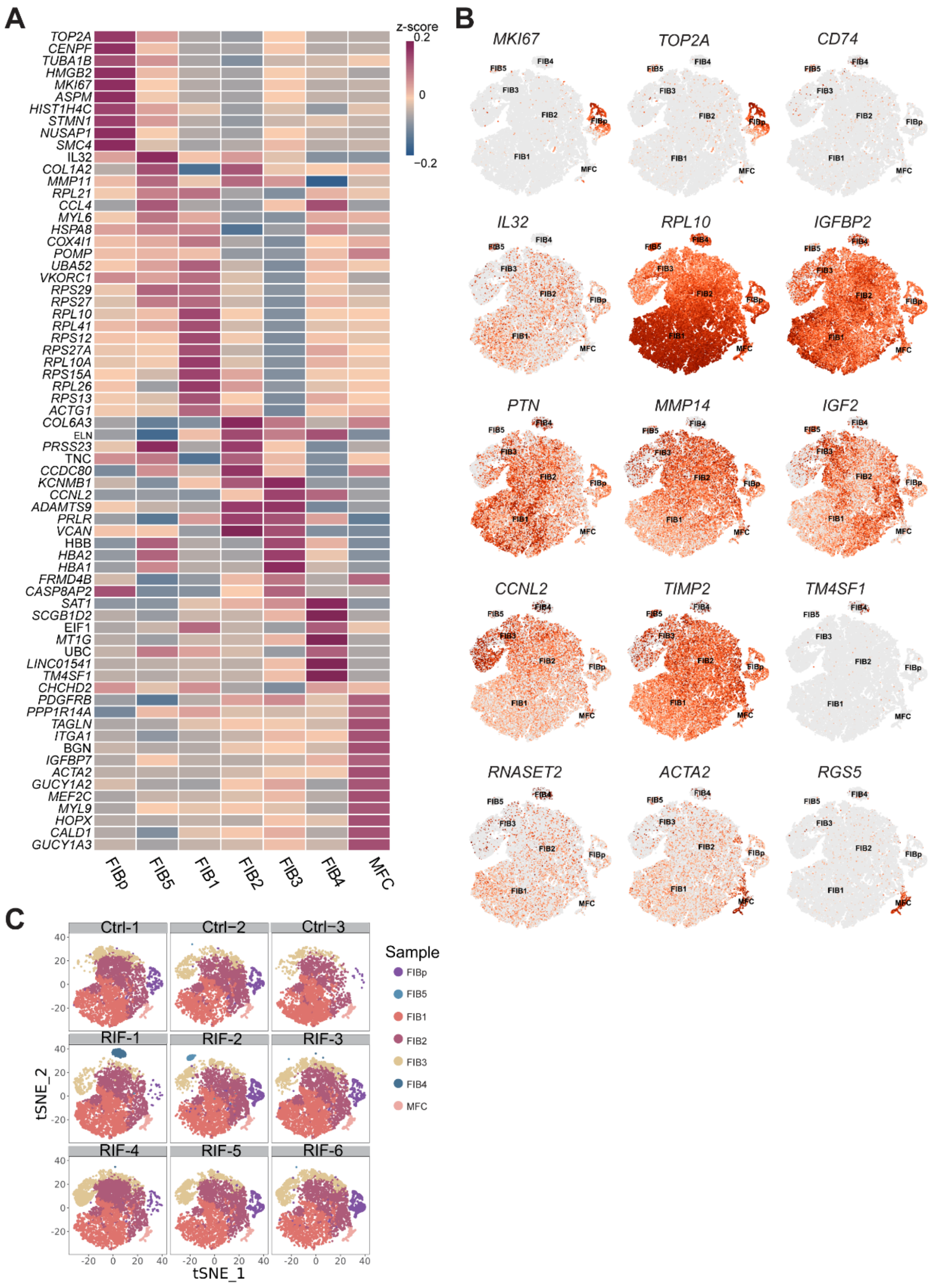

Figure S4 The subtypes and proportion of endometrial FIB from controls and RIF patients

(A) Heat map of relative expression (z-score) of selected maker genes for 7 sub-clusters of FIB

cells.

(B) Dot plots showing the expression of the indicated markers for each FIB cell cluster on the t-SNE map in Figure 2A.

(C) *t*-SNE map showing the distribution of 7 subsets of FIB in different endometrium samples (3 healthy control and 6 RIF patients).

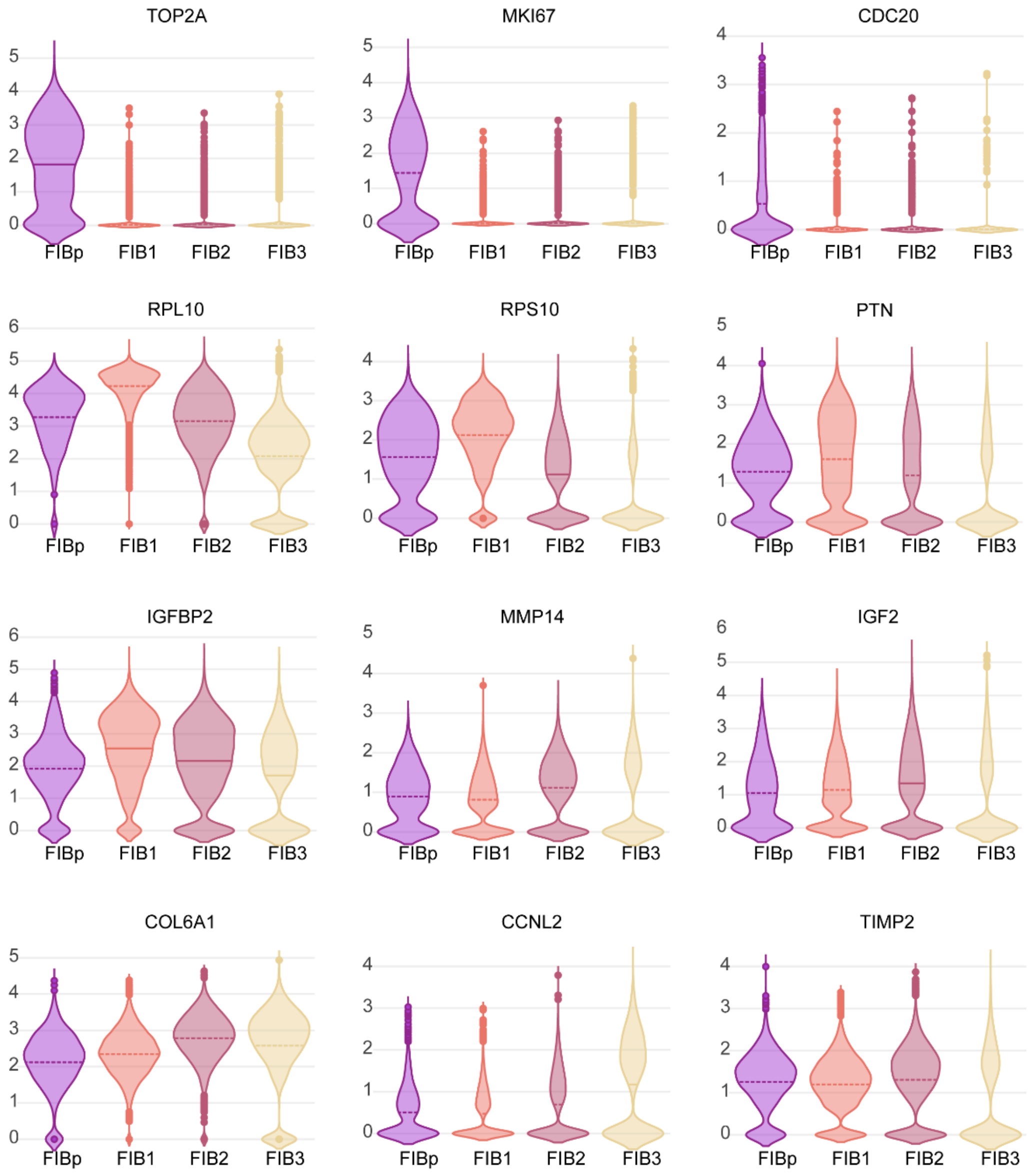

Figure S5 The mark gene expression levels of 4 FIB subsets

Violin plots showed the expression of differentially marker genes for each cluster.

A

NO.		Term ID	Term Name	P-value	(-log10P)
1	GO:MF	GO:0005102	signaling receptor binding	1.88E-04	3.725611204
2	GO:MF	GO:0019838	growth factor binding	2.98E-03	2.525201181
3	GO:MF	GO:0050840	extracellular matrix binding	6.46E-03	2.189700259
4	GO:BP	GO:0007565	female pregnancy	2.00E-08	7.698535927
5	GO:BP	GO:0042127	regulation of cell population proliferation	1.87E-06	5.728855682
6	GO:BP	GO:0035295	tube development	5.42E-06	5.266321344
7	GO:BP	GO:0060135	maternal process involved in female pregnancy	7.42E-06	5.129362037
8	GO:BP	GO:0061458	reproductive system development	1.05E-05	4.978397284
9	GO:BP	GO:0000003	reproduction	9.02E-05	4.044745317
10	GO:BP	GO:0022414	reproductive process	8.84E-05	4.053695145
11	GO:BP	GO:0046697	decidualization	4.17E-03	2.379968105
12	GO:BP	GO:0003006	developmental process involved in reproduction	5.96E-03	2.224462365
13	GO:BP	GO:0001893	maternal placenta development	1.04E-02	1.981715692
14	GO:BP	GO:0001890	placenta development	2.44E-02	1.61225434
15	GO:BP	GO:0007566	embryo implantation	4.36E-02	1.360712774
16	GO:BP	GO:0048609	multicellular organismal reproductive process	2.65E-02	1.577082019
17	GO:BP	GO:0048608	reproductive structure development	9.96E-06	5.001827878
18	GO:BP	GO:0009725	response to hormone	4.43E-03	2.354086725
19	GO:BP	GO:0032355	response to estradiol	2.19E-02	1.659555885
20	GO:CC	GO:0062023	collagen-containing extracellular matrix	4.39E-04	3.35753548

B

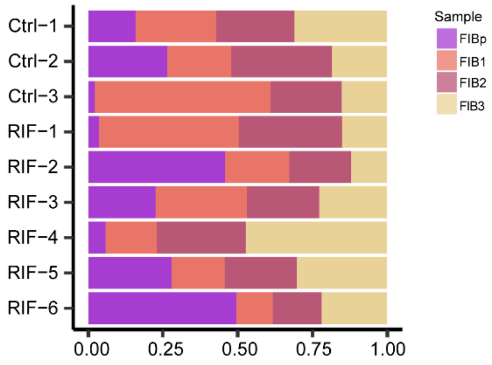

Figure S6 The marked GO terms and the proportions of 4 FIB subtypes

(A) The marked GO terms for Figure 2C, was gotten from g:Profile (https://biit.cs.ut.ee/gprofiler/).

(B) The distribution of 4 subsets of FIB in different endometrium samples (3 healthy control and 6 RIF patients).

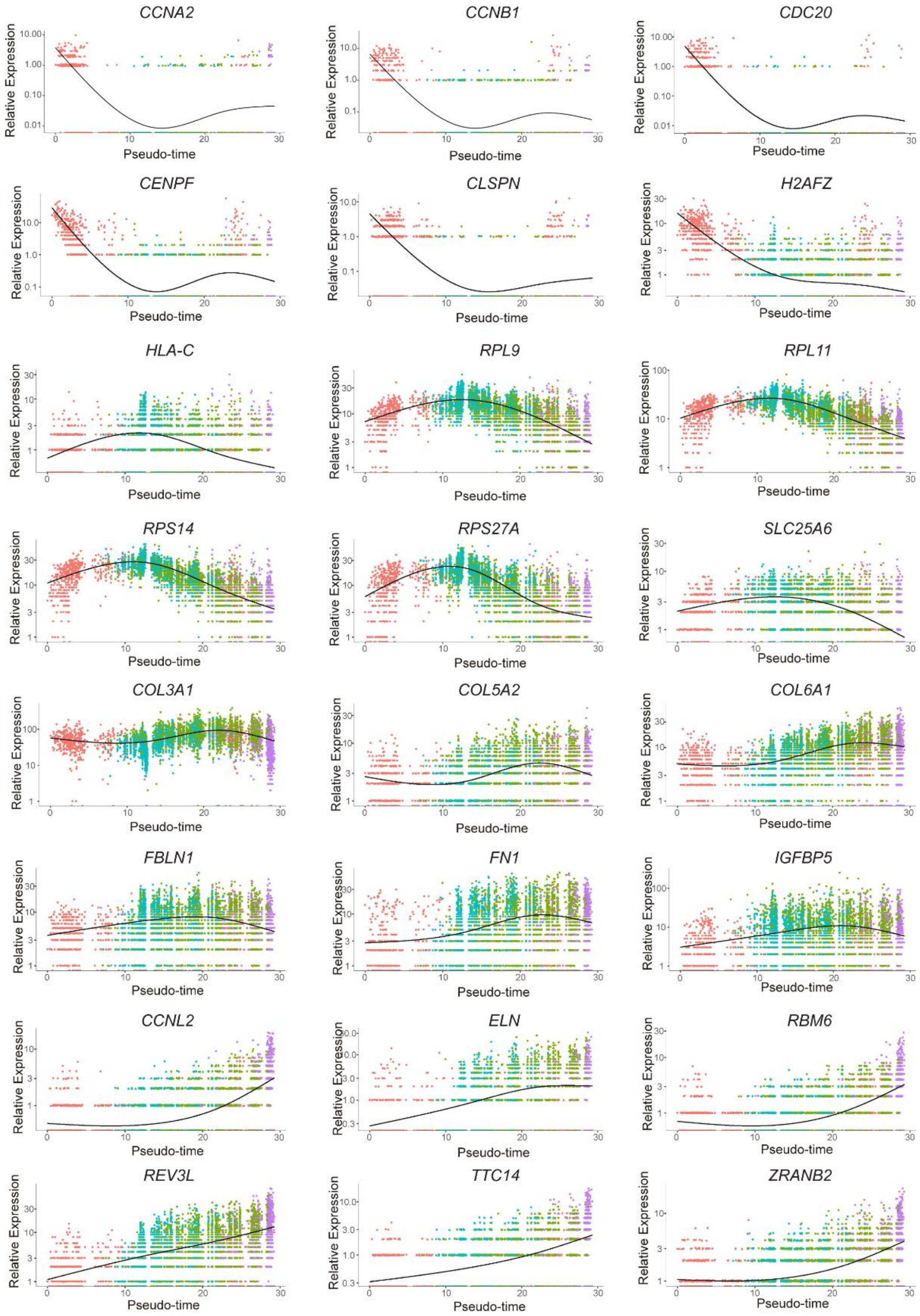

Figure S7 Expression patterns of representative genes in four ESCs along the reprogramming trajectory.

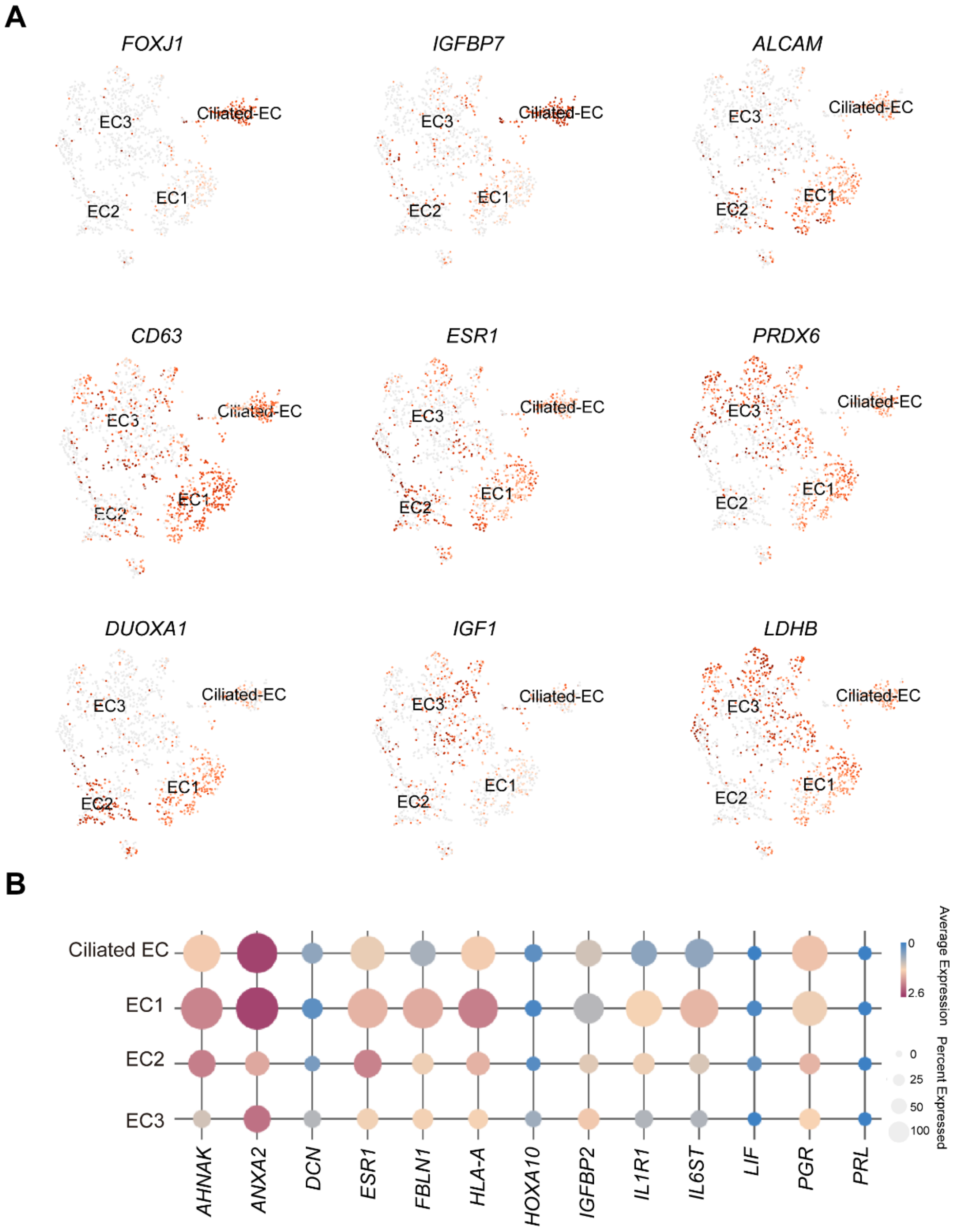

Figure S8 Characteristics of four sub-clusters of EC.

(A) Gene expression levels of marker genes across four sub-clusters of EC.

(B) Bubble diagram showing average expression of endometrial receptivity-genes for 4 sub-

clusters of EC.

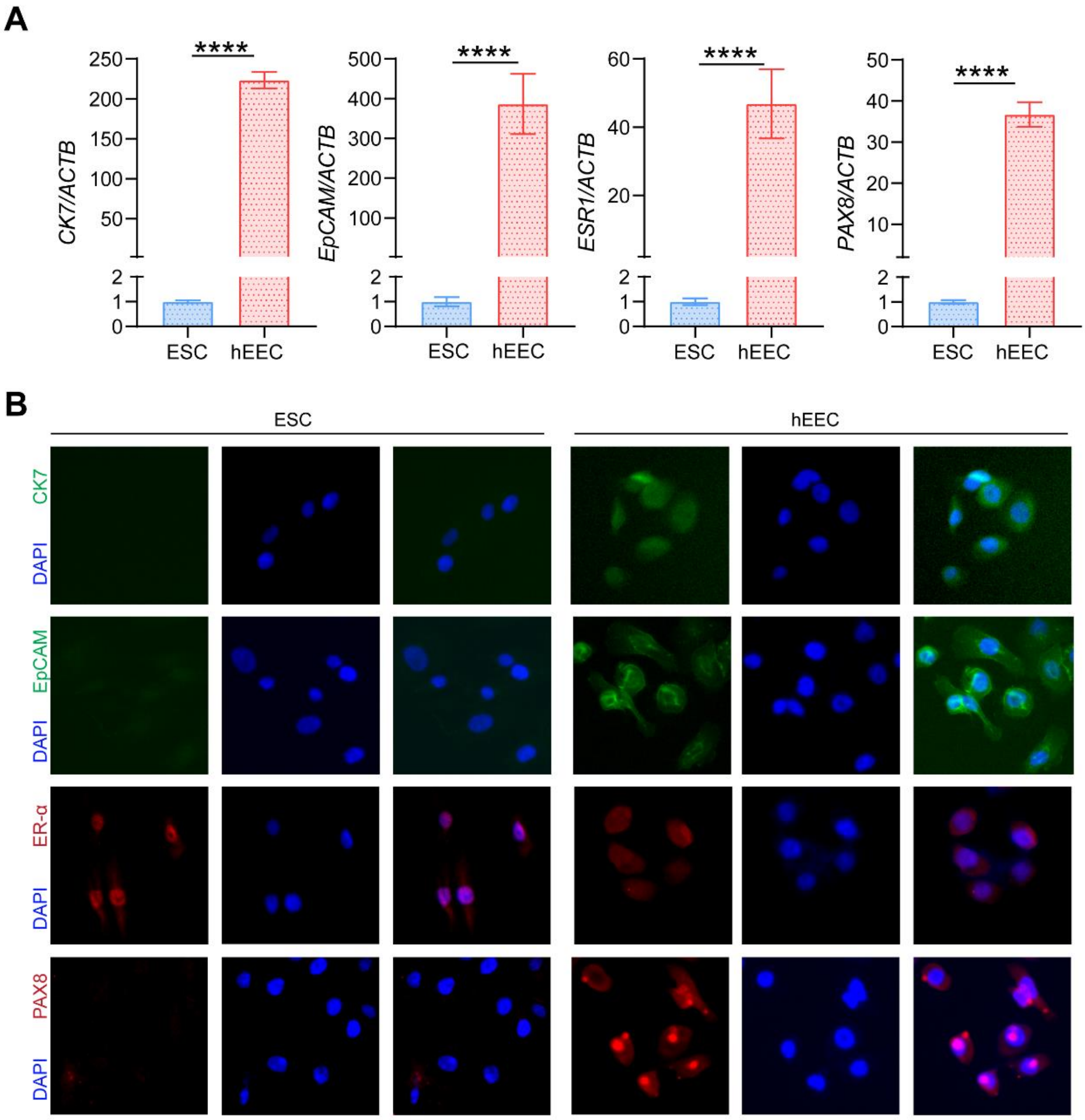

Figure S9 Characterization of the human endometrial epithelial hEEC cell line.

(A) The mRNA expression levels of *CK7*, *EpCAM*, *ESR1*, and *PAX8* were detected by RT-PCR, which were measured in hEECs and primary ESCs (n = 3). Data were presented as mean ± SEM and analyzed by t test. (****, $p < 0.0001$)

(**B**) Immunofluorescent staining of CK7, EpCAM, ER-α, and PAX8 on hEECs and primary ESCs (n = 3).

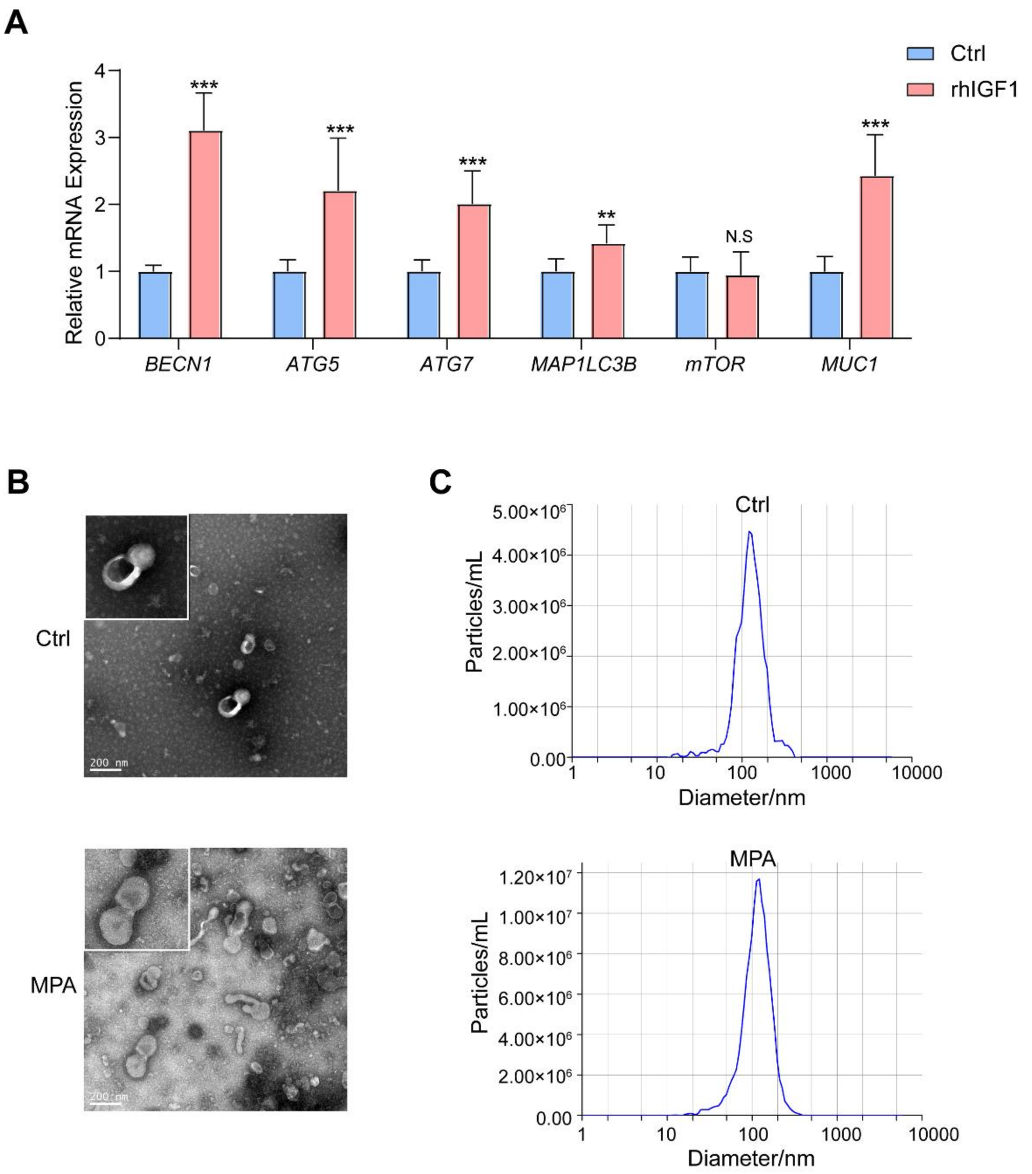

Figure S10 Progesterone increases exosomes production by EEC.

(A) HEECs were treated with rhIGF1 (2 ng/mL) or control vehicle (n = 3) for 48 h, and the mRNA expression levels of *BECN1*, *ATG5*, *ATG7*, *MAP1LC3B*, *mTOR*, and *MUC1* were

detected by RT-PCR. Data were presented as mean ± SEM and analyzed by t test. (NS, no significance, **, $p < 0.01$, ***, $p < 0.001$)

(B) Representative transmission electron microscopy (TEM) analysis from exosomes enriched from 0.1% DMEM-treated or 1 μM MPA-treated EECs. Scale bar, 200 nm.

(C) Nanoparticle tracking analysis (NTA) from enriched exosomes (0.1% DMEM-treated or 1 μM MPA-treated EECs) depicting size distribution patterns.

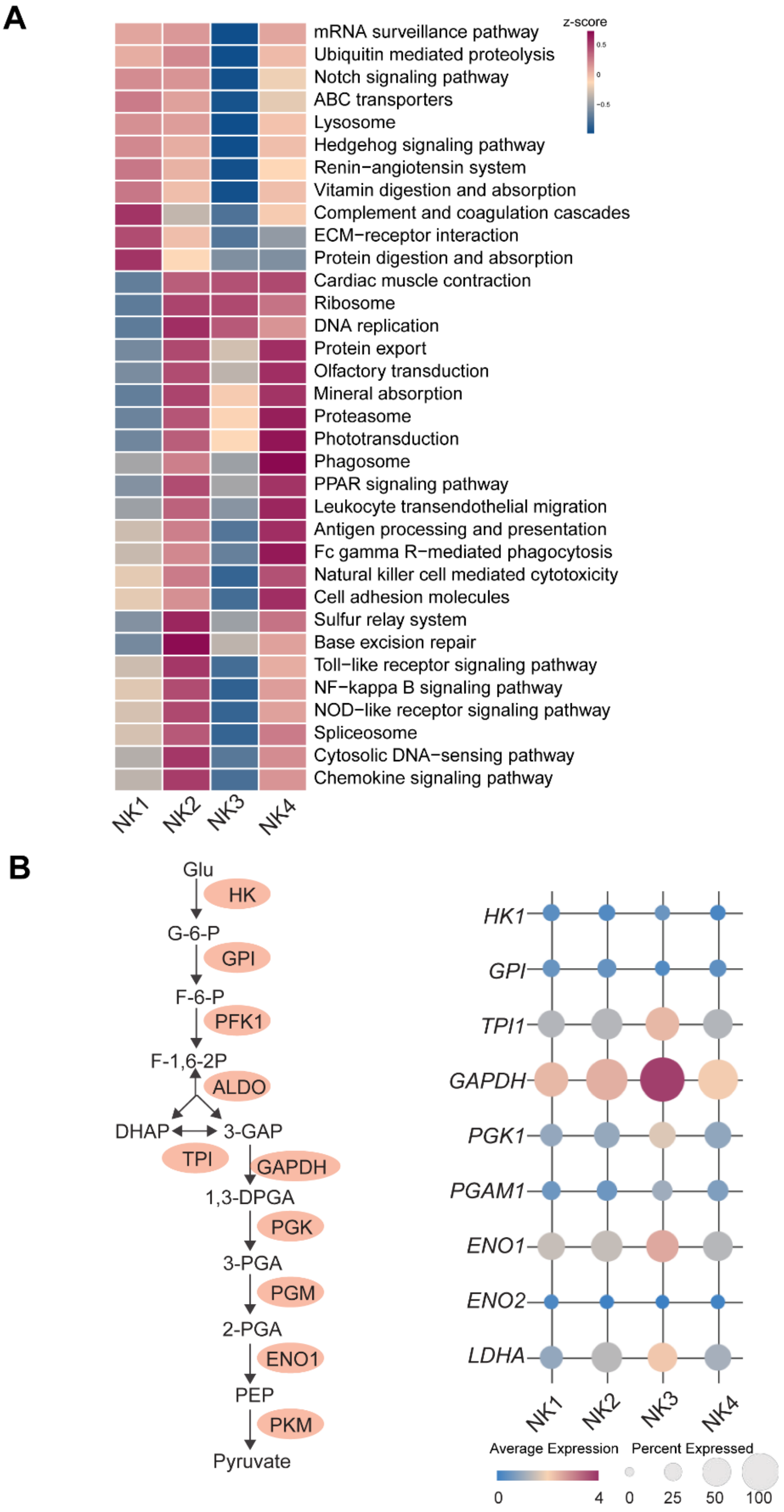

Figure S11 Characteristics of four NK subtypes.

(A) QuSAQE analysis of functional pathway in four sub-clusters of endometrial NK cells.

(B) Bubble diagram showing the expression of glycolysis-related genes in endometrial NK1,

NK2, NK3, and NK4 cells.

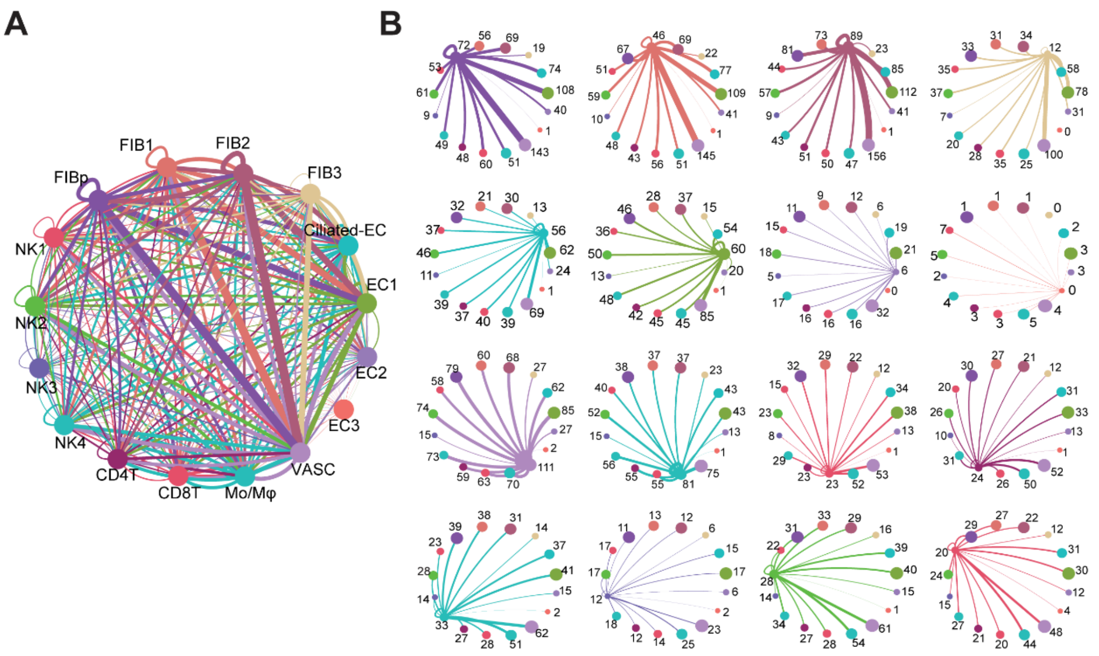

Figure S12 Intercellular communication analysis among all cell types in endometrium.

(A) Intercellular communication analysis among all cell types in endometrium. Line color indicates ligands broadcast by the cell population of the same color (labeled). Lines connect to

cell populations where cognate receptors are expressed. Line thickness is proportional to the number of ligands where cognate receptors are present in the recipient cell population. Loops indicate autocrine circuits. Map quantifies potential communication but does not account for anatomic position or boundaries of cell populations.

(B) Detailed view of ligands broadcast by each cell type and those populations expressing cognate receptors primed to receive a signal. Numbers indicate the quantity of ligand-receptor pairs for each inter-population link. Colors of the cell population corresponds to (A) above.

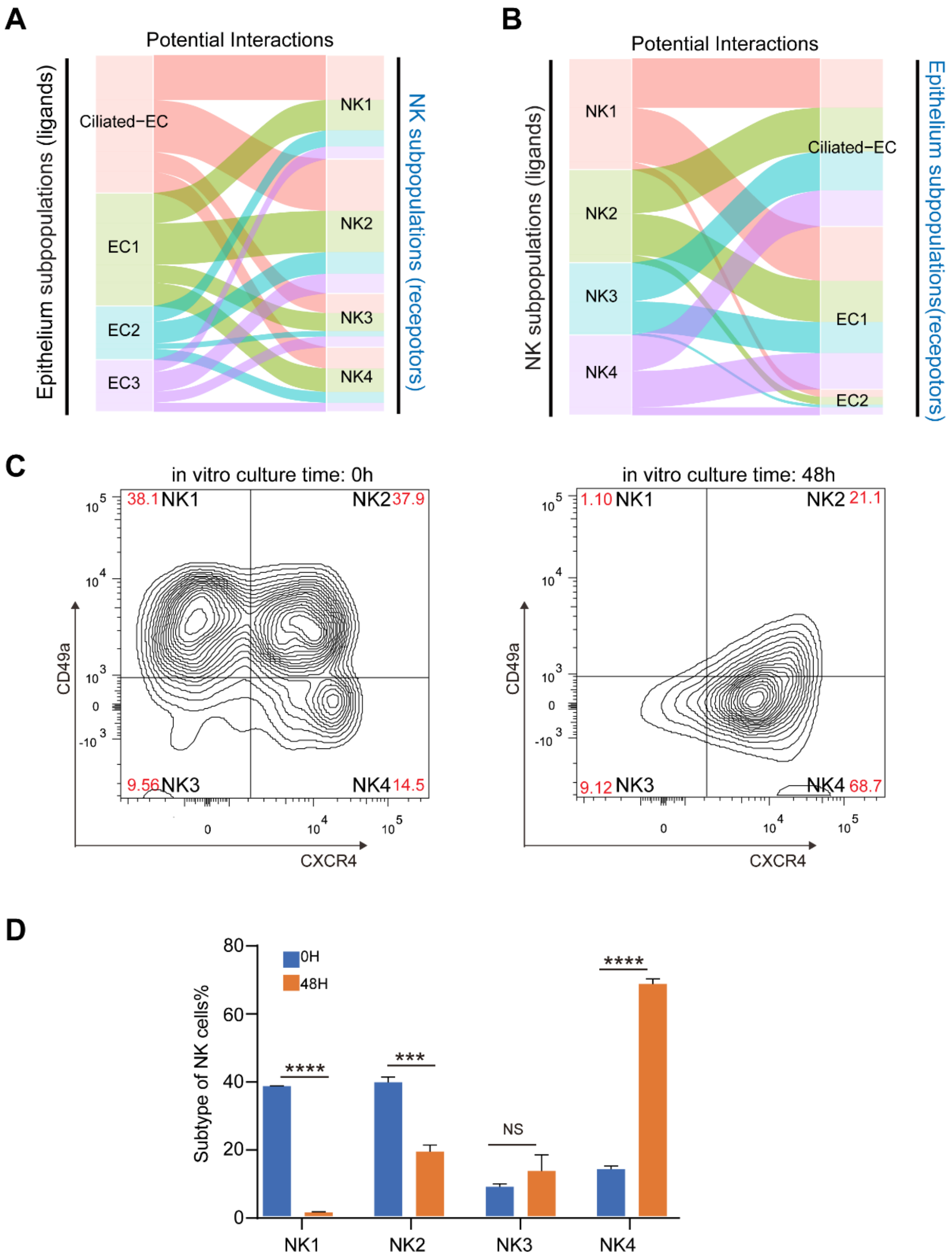

Figure S13 The potential interactions between endometrial EC and NK cells. And the proportion of four sub-clusters of NK after 48 hours *in vitro* culture.

(A, B) Potential interactions between four subpopulations of EC and NK cells based on

receptor-ligand pairs. The width of the line represented the number of receptor-ligand pairs.

(C, D) FCM was used to determine the proportions of four groups of NK cells from control group during secretory phase (n = 6 for each group) after 0- or 48-hours in vitro culture. Cells were initially gated within $CD45^+CD3^-CD56^+$ gate, and then CXCR4 and ITGA1 gating. Data were presented as mean ± SEM and analyzed by t test. (NS, no significance, ****, $p < 0.0001$)
